# Discovery of a Novel Series of Potent, Selective, Orally Available, and Brain-Penetrable C1s Inhibitors for Modulation of the Complement Pathway

**DOI:** 10.1021/acs.jmedchem.3c00348

**Published:** 2023-04-25

**Authors:** Zenichi Ikeda, Taku Kamei, Yusuke Sasaki, Matthew Reynolds, Nozomu Sakai, Masato Yoshikawa, Michiko Tawada, Nao Morishita, Douglas R. Dougan, Chien-Hung Chen, Irena Levin, Hua Zou, Masako Kuno, Naoto Arimura, Yusuke Kikukawa, Mitsuyo Kondo, Kimio Tohyama, Kenjiro Sato

**Affiliations:** † Research, Takeda Pharmaceutical Company Ltd., 26-1, Muraoka-Higashi 2-chome, Fujisawa, Kanagawa 251-8555, Japan; ‡ Structural Biology, Takeda Development Center Americas, Inc., San Diego, California 92121, United States; § Discovery Biology, Discovery Science, Axcelead Drug Discovery Partners, Inc., 26-1, Muraoka-Higashi 2-chome, Fujisawa, Kanagawa 251-0012, Japan

## Abstract

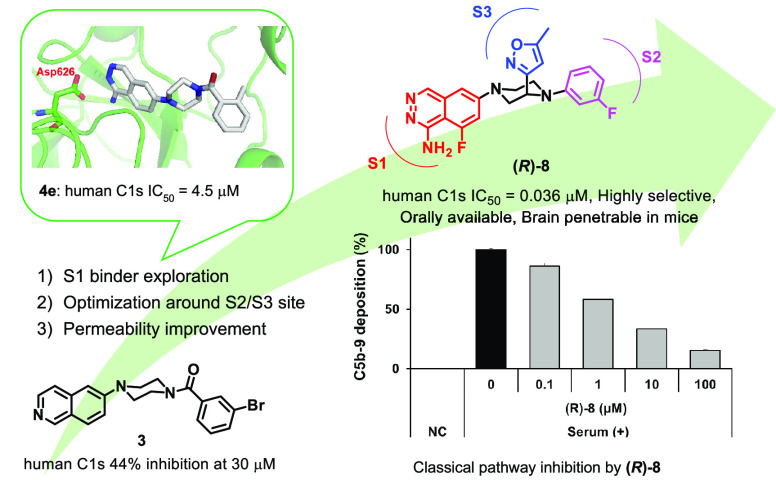

A novel series of non-amidine-based C1s inhibitors have been explored. Starting from high-throughput screening hit **3**, isoquinoline was replaced with 1-aminophthalazine to enhance C1s inhibitory activity while exhibiting good selectivity against other serine proteases. We first disclose a crystal structure of a complex of C1s and a small-molecule inhibitor (**4e**), which guided structure-based optimization around the S2 and S3 sites to further enhance C1s inhibitory activity by over 300-fold. Improvement of membrane permeability by incorporation of fluorine at the 8-position of 1-aminophthalazine led to identification of **(*R*)-8** as a potent, selective, orally available, and brain-penetrable C1s inhibitor. **(*R*)-8** significantly inhibited membrane attack complex formation induced by human serum in a dose-dependent manner in an in vitro assay system, proving that selective C1s inhibition blocked the classical complement pathway effectively. As a result, **(*R*)-8** emerged as a valuable tool compound for both in vitro and in vivo assessment.

## Introduction

The complement system is a component of the innate immune system consisting of three pathways, namely, the classical, lectin, and alternative pathways. Activation of the complement system induces phagocytosis via opsonization by C3b deposition, anaphylatoxin (C3a, C4a, and C5a) production, and C5b-9 membrane attack complex (MAC) formation to eliminate pathogens from the body in healthy individuals.^1^ On the other hand, the complement system also plays a role in various diseases. For example, impaired complement activity increases susceptibility to severe infections and also contributes to the development of autoimmune diseases,^2^ whereas overactivation of the complement pathway contributes to chronic inflammation and tissue injury.^3^ In addition, the components of the complement system are expressed in the brain as well as peripheral tissues, and it has been reported that C1q and downstream complement proteins (e.g., C3) are involved in synapse elimination in the brain, suggesting pathological roles of the complement system in central nervous system diseases as well.^4^ Because of the involvement of the complement system in various diseases, it has attracted significant attention as a target for drug development.^5^ For instance, a C1 esterase inhibitor is used for patients with hereditary angioedema, and eculizumab, a C5 inhibitor, is applied for the treatment of atypical hemolytic uremic syndrome and paroxysmal nocturnal hemoglobinuria.^6−8^ Various types of complement inhibitors are under clinical development for many other complement-mediated indications.^5^

C1s is a member of the chymotrypsin-like serine protease family, and it activates the downstream cascade of the classical pathway in the complement system.^9^ Thus, in theory, inhibition of C1s could decrease or stop the complement cascade, and therefore, C1s could serve as a novel therapeutic agent for various complement-mediated diseases.

**Figure 1:**
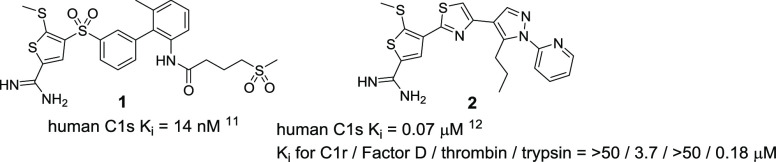
Reported small-molecule C1s inhibitors.

**Figure 2:**
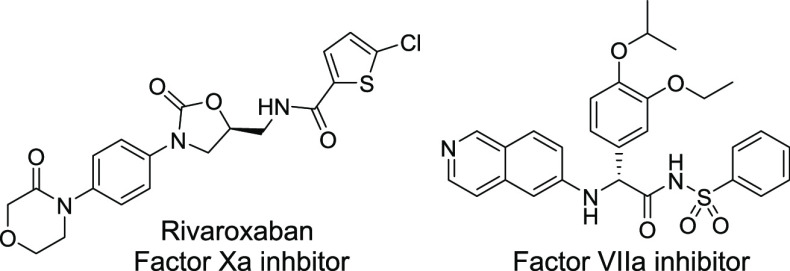
Examples of orally available serine protease inhibitors possessing non-amidine S1 binders.

C1s cleaves its substrates C4 and C2 at Arg-Ala and Arg-Lys bonds, respectively.^10^ Because one of the key recognition motifs is the ionic interaction between Asp626 in the S1 pocket of C1s and Arg of the substrate, amidines or guanidines that interact with Asp626 can act as S1 recognition motifs for C1s inhibitors. In fact, amidine derivatives, such as **1** and **2**, have been reported as potent C1s inhibitors (Figure 1).^11,12^ The docking study of a related analogue of **2** suggested that the amidine moiety interacted with Asp626 in the S1 site.^12^ Despite showing potent inhibitory activity against C1s, **1** was reported to have poor in vivo pharmacokinetics (PK) properties likely due to the basic amidine moiety.^11,13^ While a follow-up study utilizing a PEGylation strategy led to improvement of PK properties for parenteral dosing,^13^ to the best our knowledge, there are no reports on potent and orally available C1s inhibitors. On the other hand, as shown in Figure 2, several orally available serine protease inhibitors bearing a non-amidine S1 binder have been reported.^14,15^ Therefore, exploration of non-amidine-based compounds was considered a promising approach to discover potent and orally available C1s inhibitors.

Regarding selectivity against other serine proteases, **2** showed excellent selectivity against C1r, a serine protease located upstream of C1s. However, there is room for improvement on the selectivity against other serine proteases, including the related complement cascade protease (e.g., factor D) and other general serine proteases (e.g., trypsin).^12^ We considered that it might be difficult to secure excellent selectivity among serine proteases while an amidine was utilized as an S1 binder against C1s. This motif is used to acquire high affinity via ionic interactions and thus relies on strong basicity. Thus, it was hypothesized that discovery of a non-amidine S1 binder would be crucial to eventually achieve extremely high selectivity.

Recently, non-amidine-based small-molecule C1s inhibitors were disclosed,^16−18^ although the detailed properties, such as PK properties and selectivity, are unknown. We have separately performed medicinal chemistry research without such information, and herein, we describe the discovery of a novel series of potent, selective, orally available, and brain-penetrable C1s inhibitors.

## Results and Discussion

**Figure 3:**
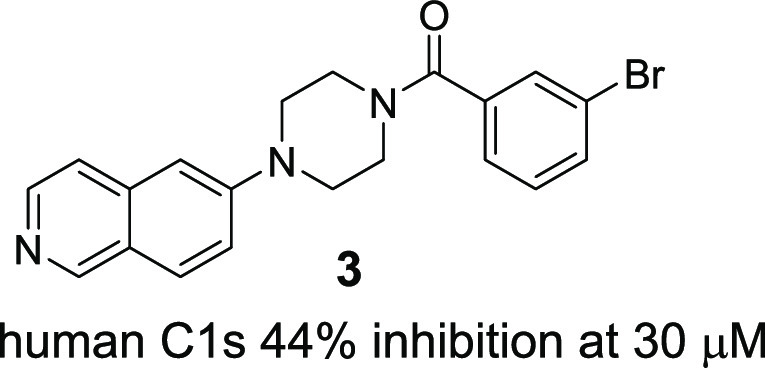
Structure of hit compound **3**.

With the aim of acquiring a promising starting point for pursuing non-amidine-based C1s inhibitors with high lead-likeness, various approaches have been implemented, such as an enzymatic assay-based high-throughput screening (HTS), an affinity selection with a DNA-encoded library, and an NMR-based fragment screening. As a result, there were few tractable and viable hits. Among them, although its C1s inhibitory activity was weak, **3** was identified as a non-amidine-based hit compound (Figure 3), which encouraged us to initiate medicinal chemistry research to identify a novel series of potent, selective, orally available, and brain-penetrable C1s inhibitors.

**Figure 4:**
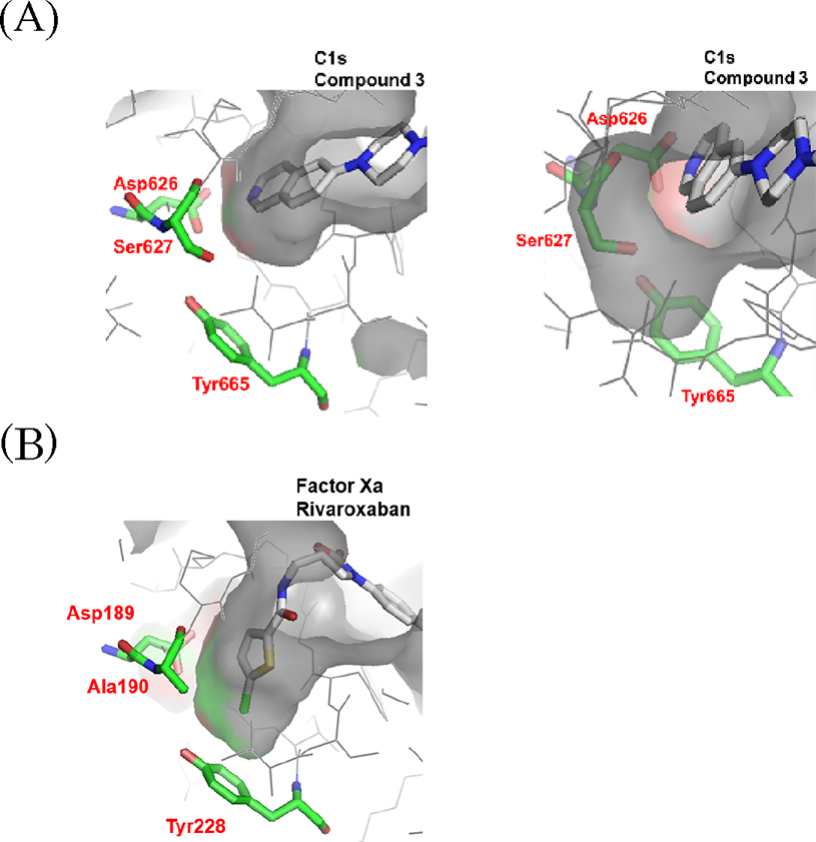
Comparison of the S1 pocket. (A) C1s (docking model with **3** based on the apo form of C1s (PDB ID: 1ELV), left: side view; right: top view). The amino acid residue numbering is aligned with UniProt (P09871). (B) Factor Xa (cocrystallized with rivaroxaban (PDB ID: 2W26)).

We constructed a docking model of **3** according to the reported X-ray crystal structure of the apo form of human C1s (PDB ID: 1ELV;^19^Figure 4A). The model indicated that the isoquinoline of **3** should act as an S1 binder to make an ionic interaction with Asp626. To enhance affinity in the S1 site, we developed two strategies: (1) acquisition of an additional interaction and (2) enhancement of the interaction with Asp626. We first considered if acquisition of other interactions at the S1 site could be possible. Aside from an ionic interaction between a basic moiety and an aspartic acid residue in the S1 site, acquisition of a Cl−π interaction between a chlorine atom and a tyrosine residue has been reported as an effective approach to discover orally available serine protease inhibitors, as exemplified by orally active factor Xa inhibitors (Figure 2). As shown in Figure 4B, Tyr228 is located deeper in the S1 pocket than Asp189, and the factor Xa inhibitor rivaroxaban interacts with Tyr228 via the Cl−π interaction.^14^ Because C1s also has the corresponding tyrosine residue (Tyr665), the Cl−π interaction acquisition is an attractive approach to design potent and orally available C1s inhibitors. However, a comparison between factor Xa and C1s revealed a key structural difference in the S1 site. As shown in Figure 4A (left), in the case of C1s, ligands appear unable to access Tyr665 due to steric repulsion with the Ser627 residue (Ala190 residue in the corresponding site of factor Xa), which indicates that it should be difficult to utilize the Cl−π interaction for design of C1s inhibitors. Subsequently, we focused on enhancement of affinity with Asp626. Regarding the interaction with Asp626, **3** shows a monodentate binding mode. Acquisition of an additional interaction through a bidentate binding mode could be expected to enhance affinity to C1s, and the docking model of **3** suggested that there is spatial room for a substituent to acquire an additional interaction with Asp626 (Figure 4A (right)). Therefore, we initiated modification of the isoquinoline of **3** to achieve a bidentate interaction with Asp626.

**Figure 5:**
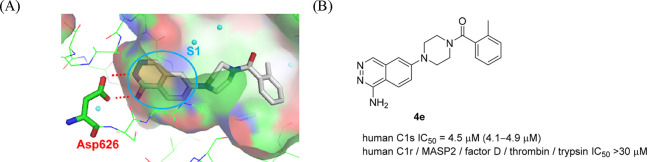
(A) Crystal structure of **4e** in complex with human C1s (PDB ID: 8GMN). (B) Chemical structure and in vitro activity of **4e**. IC_50_ values are presented with their 95% confidence intervals in parentheses.

The inhibitory activity of each synthesized compound against human C1s was evaluated. As shown in Table 1, incorporation of an amino moiety at the 1-position of the isoquinoline to furnish 1-aminoisoquinoline was not effective (**4b**). However, replacement of the 1-aminoisoquinoline with 1-aminophthalazine dramatically enhanced C1s inhibitory activity by around 40-fold (**4c**). Interestingly, removal of the 1-amino moiety of **4c** to afford a phthalazine resulted in a remarkable drop in potency (**4d**) with much weaker potency than **4a**, which demonstrated that the combination of the phthalazine scaffold and the amine moiety is crucial to exhibit potent C1s inhibitory activity. On the basis of the calculated p*K*_a_ value of each compound, it was considered that a certain level of basicity was essential to inhibit C1s (**4a** vs **4d**) and that **4c** likely got a bidentate interaction with Asp626 to enhance C1s inhibitory activity. On the other hand, it is difficult to explain the difference in IC_50_ values between **4b** and **4c** only by basicity. To better understand the structural requirement of the 1-aminophthalazine moiety, we obtained crystal structural information of a 1-aminophthalazine **4e** in complex with C1s, which, to the best of our knowledge, was the first report of a cocrystal structure between human C1s and a small-molecule ligand. As shown in Figure 5A, the structure revealed that the amino moiety at the 1-position of the phthalazine ring and the nitrogen atom at the 2-position made a bidentate interaction with Asp626 in the S1 pocket of C1s as expected, thereby boosting potency. Furthermore, the nitrogen atom at the 3-position of the phthalazine ring does not undergo hydrogen bonding with the C1s protein directly and possibly plays a role in facilitating improved penetration into the S1 pocket compared to the 1-aminoisoquinoline (**4b**). In addition to potent C1s inhibitory activity, **4e** showed good selectivity against not only C1r, MASP-2, and factor D, serine proteases involved in the complement pathway, but also other serine proteases such as thrombin and trypsin (IC_50_ > 30 μM, respectively) (Figure 5B). As benzamidine shows high basicity (p*K*_a_ = 11.6),^20^ it was expected that these weakly basic non-amidine compounds could be a good starting point to pursue selective and orally available C1s inhibitors. Thus, we identified **4c** and **4e** as novel attractive lead compounds of selective C1s inhibitors possessing the 1-aminophthalazine moiety as a unique non-amidine-based S1 binder.

**Table 1:**
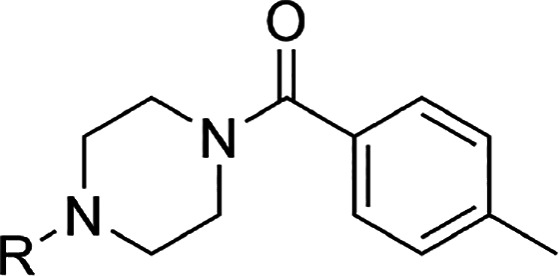

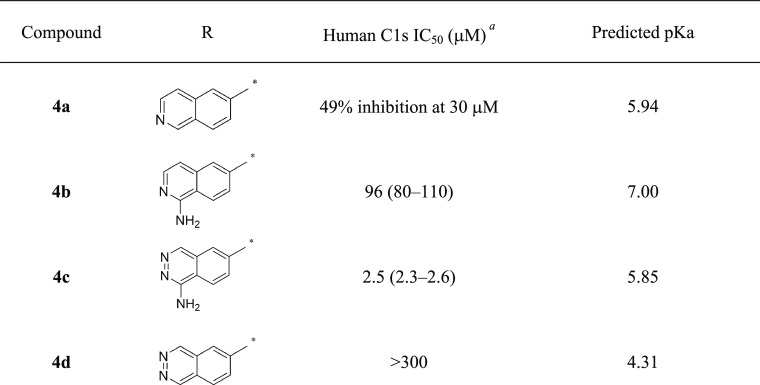
In Vitro Activities of **4a**–**d**

a Inhibitory activity of compounds against human C1s. IC_50_ values are presented with their 95% confidence intervals in parentheses.

**Figure 6:**
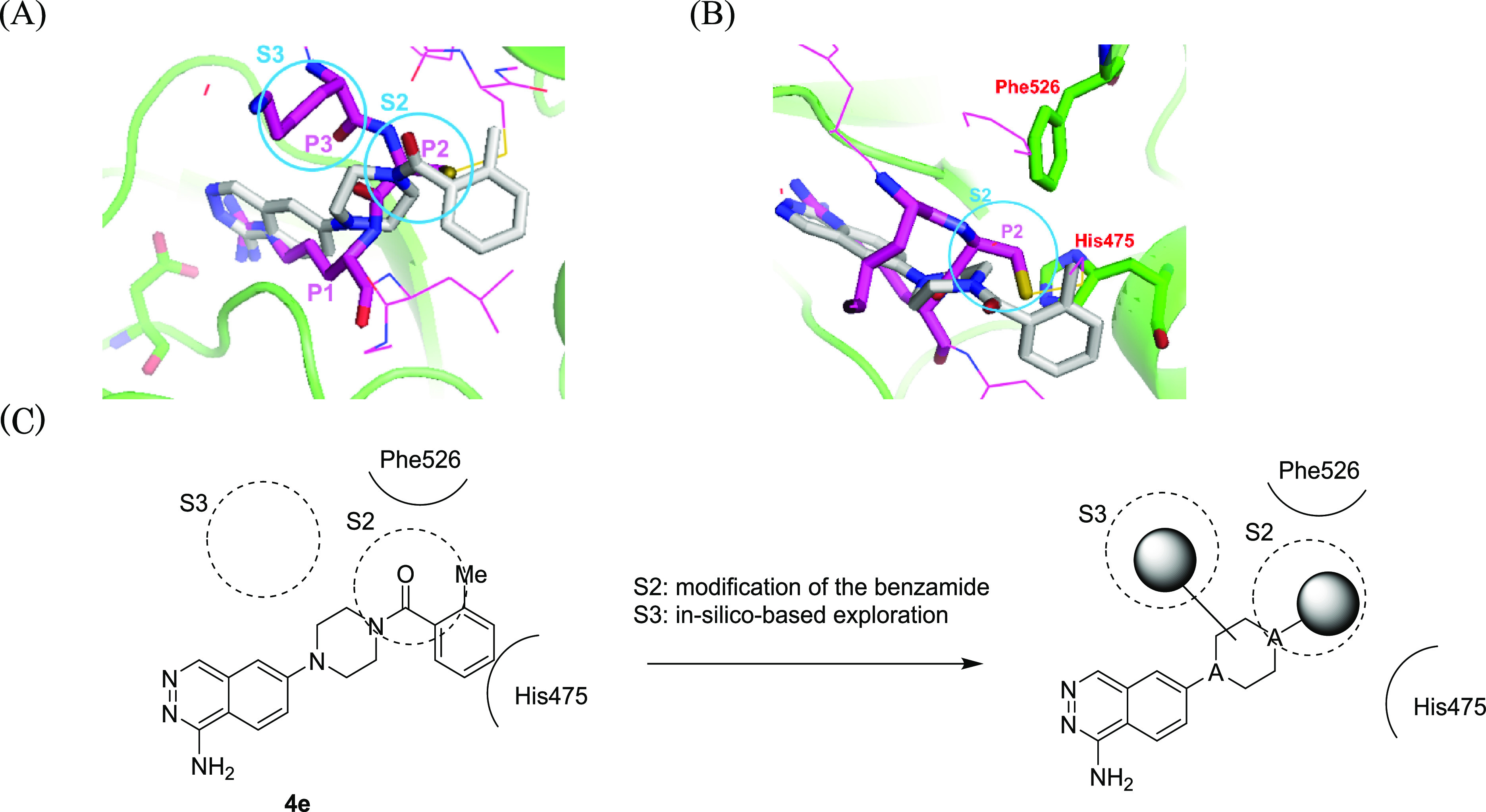
(A) Superposition of gigastasin (PDB ID: 5UBM, magenta) with the **4e** (white)–C1s (green) complex (PDB ID: 8GMN). P1 (Lys65), P2 (Cys64), and P3 (Lys63) of gigastasin are shown as sticks. (B) Top view around the S2 site. (C) Strategy to enhance C1s inhibitory activity.

On the basis of the binding pose of **4e** cocrystallized with C1s, a structure-based approach was performed to further enhance potency. Figure 6A shows the superposition of gigastasin,^21^ a peptidic C1s/MASP-2 inhibitor, with the **4e**-C1s complex. It has been reported that gigastasin occupies the S2 and S3 site of C1s through interaction with Cys64 (P2) and Lys63 (P3), respectively. In the case of **4e**, the amide moiety was located around the S2 site. Because the S2 site is a lipophilic environment surrounded by His475 and Phe526 (Figure 6B), **4e** is not likely to occupy the S2 site effectively. Furthermore, there are no substituents of **4e** located in the S3 site. Therefore, enhancement of affinity in the S2 site and acquisition of a novel interaction in the S3 site were attractive targets for enhancement of C1s inhibitory activity. As shown in Figure 6C, it was hypothesized that the migration of the lipophilic benzene ring of **4e** closer to the piperazine motif would enhance the affinity via effective utilization of the S2 site. Regarding the S3 site, an in silico approach was performed to identify novel interactions effectively.

**Figure 7:**
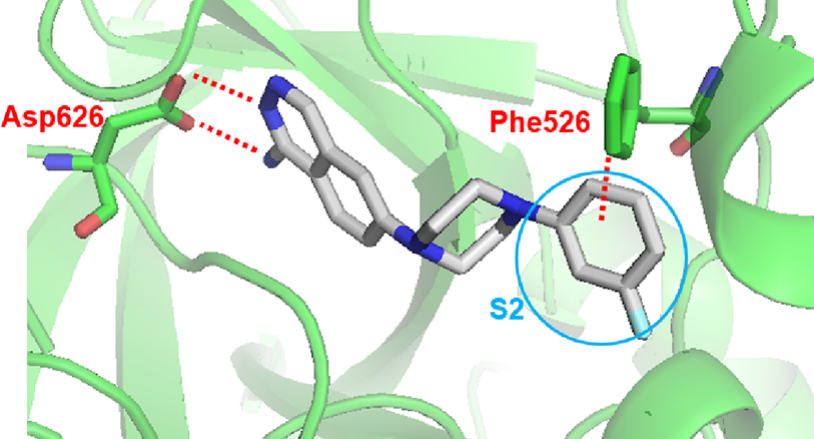
Docking model of **5g** based on the **4e**–C1s complex (PDB ID: 8GMN).

As shown in Table 2, removal of the carbonyl moiety of **4f** resulted in the enhancement of C1s inhibitory activity (**5a**). Replacement of the nitrogen atom attached to the terminal benzene ring with a carbon atom decreased potency (**5b**). Furthermore, the regioisomer of the piperidine analogue **5c** showed dramatically reduced potency, which indicated that the nitrogen atom attached to the 1-aminophthalazine ring plays an important role for C1s inhibition via an electron-donating effect to make the 1-aminophthalazine more basic and/or a conformational effect to occupy the S1 and S2 site exquisitely. As a result, among **5a**–**5c**, phenylpiperazine **5a** was considered as the optimum moiety for effective C1s inhibition. Next, we optimized around the terminal benzene ring. A methyl scan indicated that the meta-position was well-tolerated for substituent installation (**5d**–**5f**). Regarding this meta-position, incorporation of a fluoro or chloro moiety (**5g** and **5h**) also resulted in comparable potency to **5a**, whereas introduction of polar moieties such as methoxy or cyano led to a decrease in potency (**5i** and **5j**), suggesting that lipophilic substituents should be favorable in this area, surrounded by His475 and Phe526. To investigate if the terminal benzene effectively occupies the S2 site, a docking study was performed with **5g**. As shown in Figure 7, the terminal benzene ring was accommodated in the S2 site and predicted to make a CH−π interaction with Phe526, which likely contributed to enhancement of C1s inhibitory activity. The piperazine moiety of **5g** was thought to be a suitable linker to allow the key pharmacophores, the 1-aminophthalazine moiety and the terminal benzene ring, to adopt the optimal spatial configuration. Thus, we found the advanced lead series with submicromolar IC_50_ values, and **5g** was selected for further chemical modification.

**Table 2:**
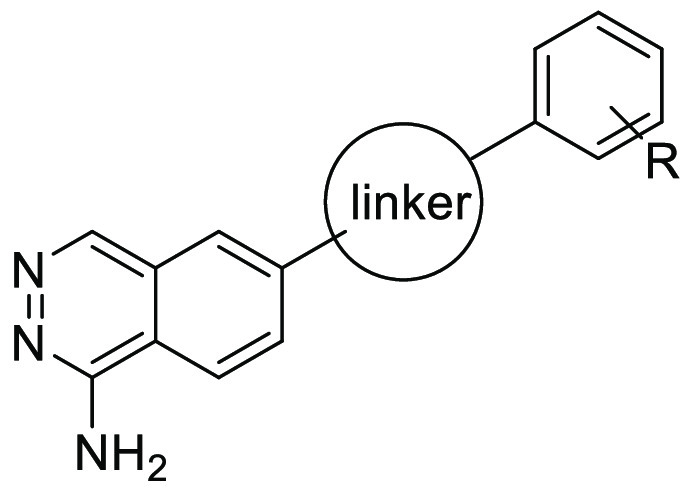

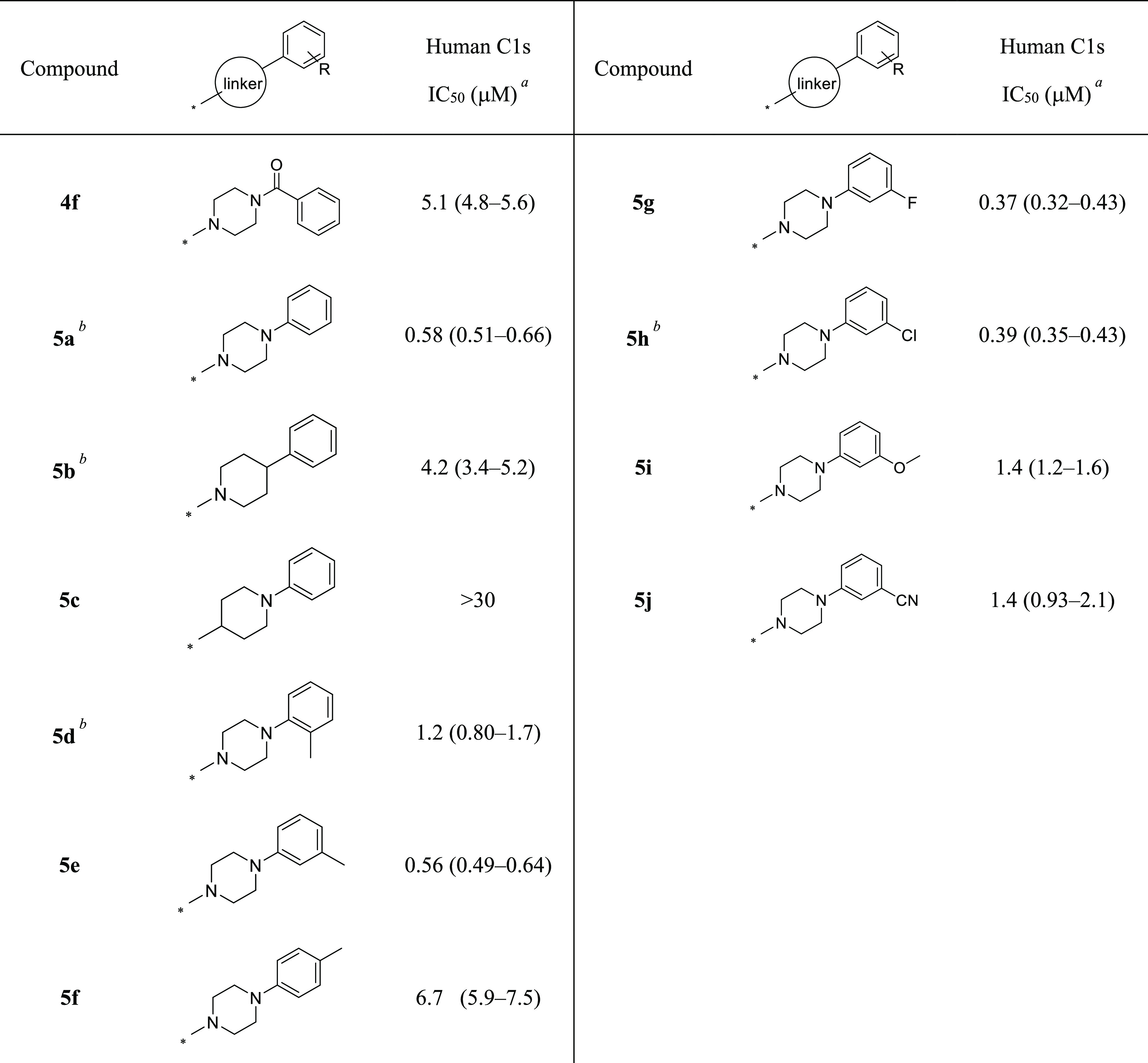
In Vitro Activities of **4f** and **5a**–**j**

a Inhibitory activity of compounds against human C1s. IC_50_ values are presented with their 95% confidence intervals in parentheses.

b TFA salt.

**Figure 8:**
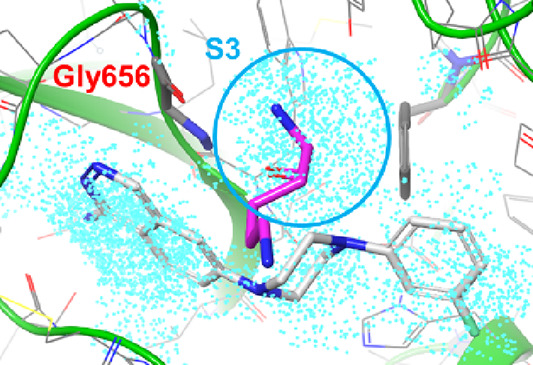
Hot spot prediction based on the **4e**–C1s complex (PDB ID: 8GMN). Predicted hot spots are shown with cyan dots. The plausible binding pose of **5g** was superposed. P3 (Lys63) of gigastasin (PDB ID: 5UBM) is shown by magenta sticks.

**Figure 9:**
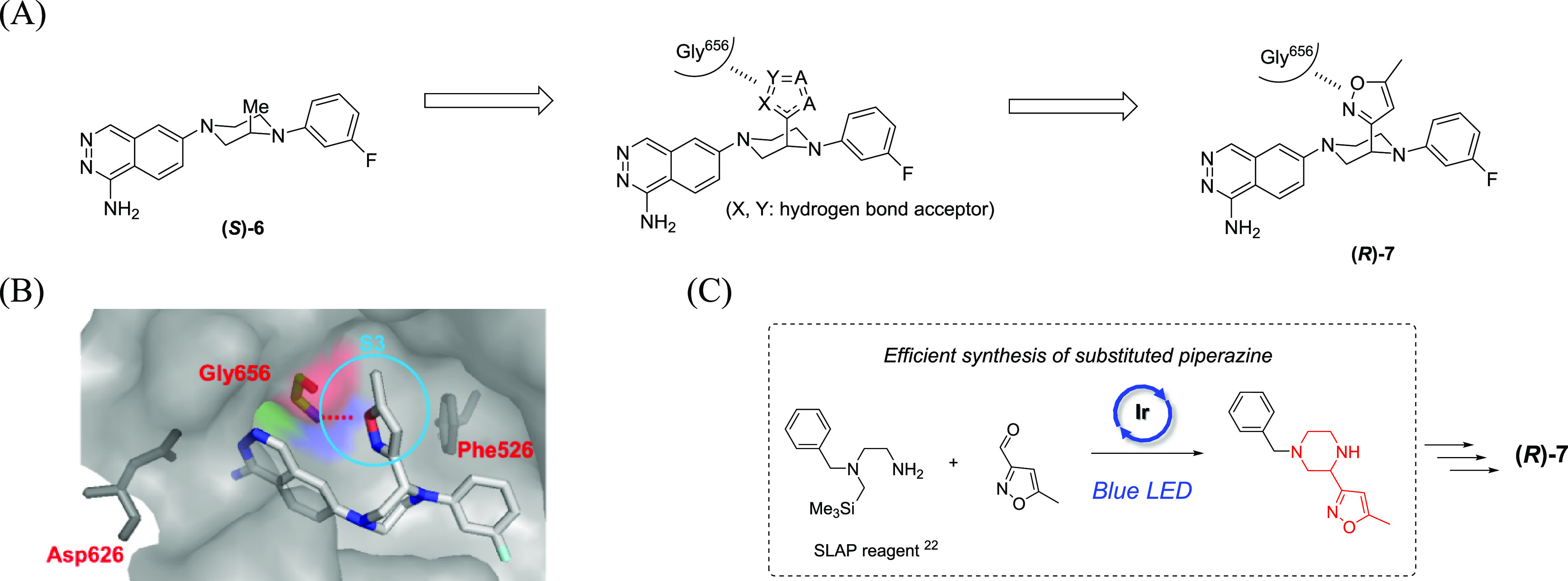
(A) Design concept to obtain additional interactions in the S3 site. (B) Predicted binding mode of **(*R*)-7** based on the **4e**–C1s complex (PDB ID: 8GMN). (C) Synthetic strategy for the key intermediate. The substructure shown in red is incorporated in **(*R*)-7**.

To occupy the S3 site effectively, an in silico-based hot spot prediction was performed. As shown in Figure 8, presumed hot spots were found in the deep region of the S3 site, occupied by the main chain of Lys63 (P3) of gigastasin. To access that region from **5g**, we considered that an axial substituent at the 3-position of piperazine should be optimal. As shown in Table 3, among the two enantiomerically pure 3-Me analogues, **(*S*)-6** was found to enhance potency over **5g**, which confirmed the validity of the approach for the S3 site. Furthermore, the main chain of Gly656 of C1s was found in close proximity to the predicted hot spots, suggesting that C1s inhibitory activity could be enhanced by acquisition of hydrogen bonding in the S3 site while keeping the active conformation of **5g**. To obtain an additional interaction, incorporation of a heteroaromatic ring possessing nitrogen and/or oxygen atoms should be a suitable approach, and thus, we designed the isoxazole derivative **(*R*)-7** (Figure 9A). The predicted binding mode of **(*R*)-7** (Figure 9B) supported the hypothesis, demonstrating that the oxygen and/or nitrogen atom of the isoxazole ring could interact with the NH of Gly656. In addition, the methyl moiety at the 5-position of the isoxazole ring exists in close proximity to the C1s wall, which can be expected to acquire an additional lipophilic interaction. As a synthetic strategy, we applied photoredox chemistry with silicon amine protocol (SLAP) reagents to furnish the key substituted-piperazine moiety efficiently (Figure 9C).^22^

**Table 3:**
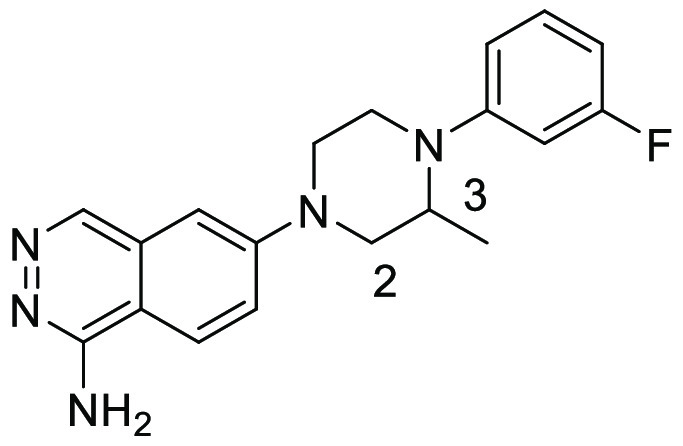

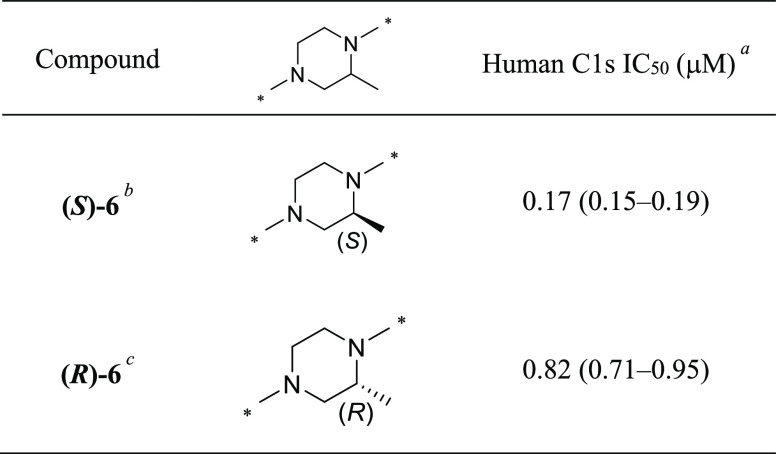
In Vitro Activities of **(*S*)-6** and **(*R*)-6**

a Inhibitory activity of compounds against human C1s. IC_50_ values are presented with their 95% confidence intervals in parentheses.

b (*S*)-Configuration at the 3-position of piperazine.

c (*R*)-Configuration at the 3-position of piperazine.

As shown in Table 4, introduction of the 5-methyl-isoxazole ring dramatically enhanced C1s inhibitory activity (**(*R*)-7**), indicating that the 5-methyl-isoxazole moiety effectively occupied the S3 site. On the other hand, it was revealed that membrane permeability of **(*R*)-7** was suboptimal (Table 4). As the 1-aminophtalazine moiety, possessing two hydrogen bond donors, was thought to contribute to poor permeability, a fluorine atom was incorporated at the 8-position of the aminophthalazine ring to cap one of the hydrogen bond donors via intramolecular hydrogen bonding. As a result, **(*R*)-8** showed dramatically improved permeability (Table 4) while showing potent C1s inhibitory activity with an IC_50_ value of 36 nM.

**Table 4:**
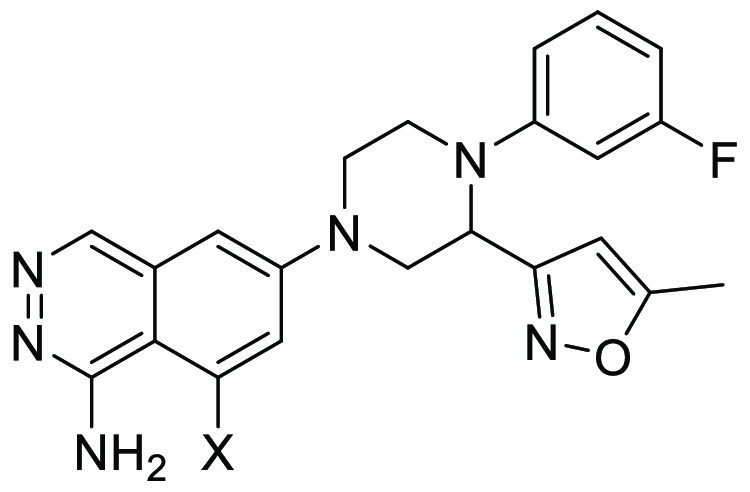

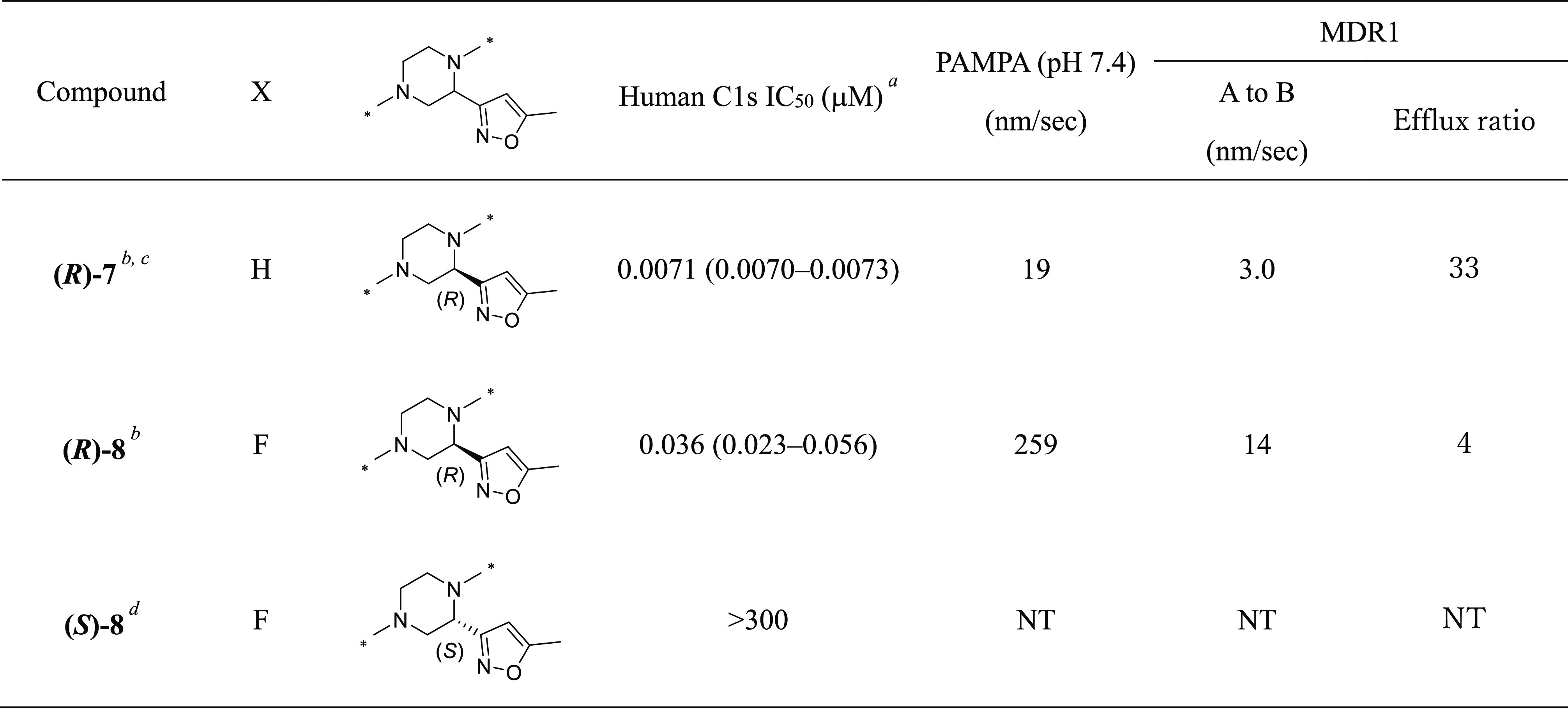
In Vitro Activities of **(*R*)-7**, **(*R*)-8**, and **(*S*)-8**

a Inhibitory activity of compounds against human C1s. IC_50_ values are presented with their 95% confidence intervals in parentheses.

b (*R*)-Configuration at the 3-position of piperazine.

c TFA salt.

d (*S*)-Configuration at the 3-position of piperazine.

**Figure 10:**
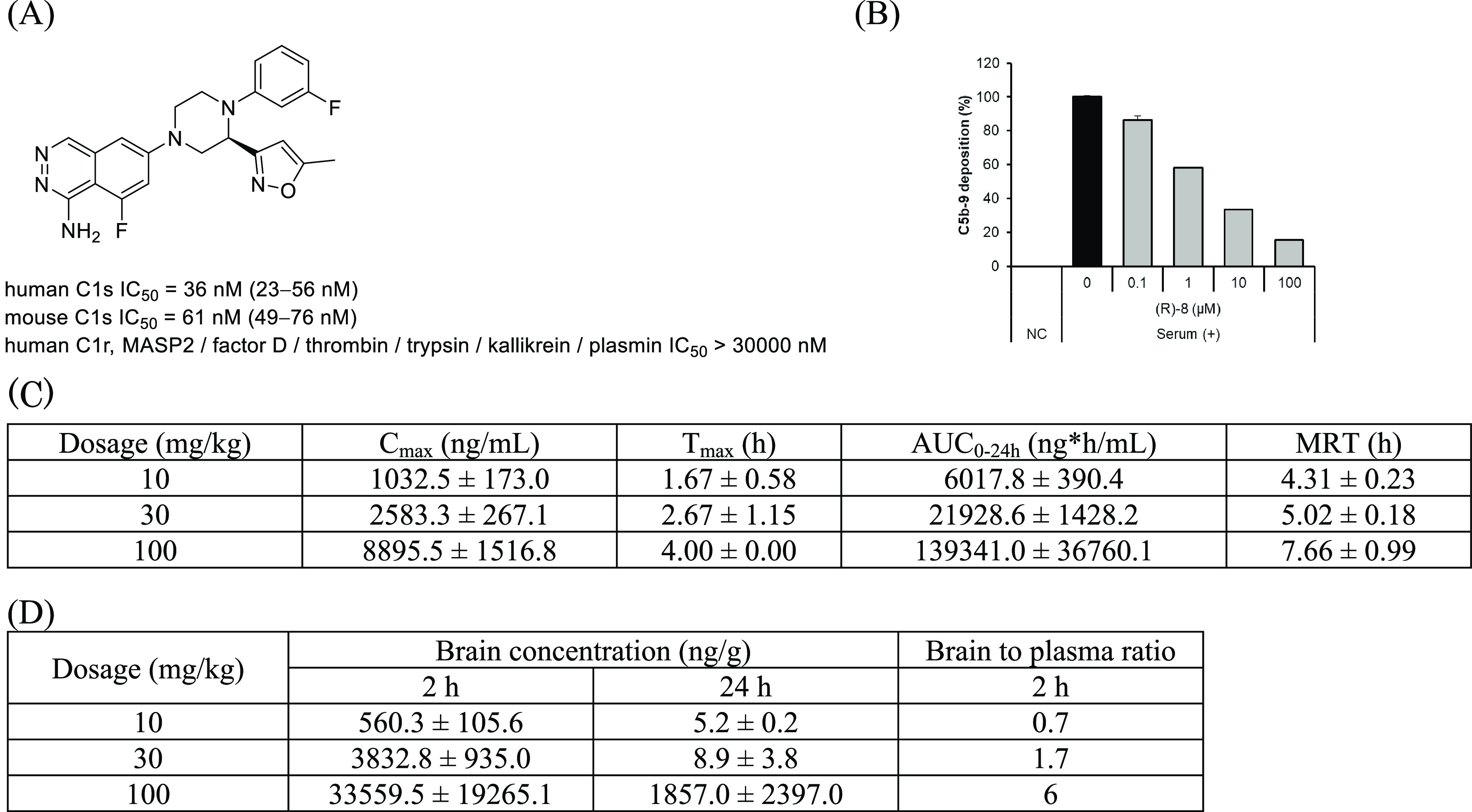
(A) Properties of **(*R*)-8**. IC_50_ values are presented with their 95% confidence intervals in parentheses. (B) In vitro assessment of **(*R*)-8**. (C) Plasma pharmacokinetics after oral administration of **(*R*)-8** in mice. (D) Brain distribution after oral administration of **(*R*)-8** in mice.

The properties of **(*R*)-8** are shown in Figure 10A. Compound **(*R*)-8** showed potent inhibitory activity against both human and mouse C1s. As for selectivity against other serine proteases, **(*R*)-8** showed excellent selectivity over C1r, MASP2, factor D, thrombin, trypsin, kallikrein, and plasmin, demonstrating that there was no compromise on selectivity in the course of optimization from the lead **4e**. As shown in Figure 10B, **(*R*)-8** significantly inhibited MAC formation induced by human serum in a dose-dependent manner, which proved that selective inhibition of C1s could effectively block the classical complement pathway. As indicated in Figure 10C, **(*R*)-8** showed plasma exposure after oral administration in mice (10, 30, and 100 mg/kg) in a dose-dependent manner. Furthermore, **(*R*)-8** was also distributed into the brain (Figure 10D) after oral administration. Collectively, **(*R*)-8** could emerge as a valuable tool compound for in vivo assessment by modulating the classical complement pathway.

In summary, we have explored non-amidine C1s inhibitors and discovered 1-aminophtalazine as a unique S1 binder to C1s. The following structure-based approach based on the crystal structure of **4e**–C1s led to the identification of a novel series of potent and selective C1s inhibitors. Compound **(*R*)-8**, showing potent C1s inhibitory activity with excellent selectivity against other serine proteases, was shown to effectively inhibit MAC formation in the in vitro assay system. Furthermore, **(*R*)-8** was found to be orally available and brain-penetrable in mice. Taken together, **(*R*)-8** was identified as a valuable in vitro and in vivo tool compound of C1s inhibitors. We believe that this approach, starting from the investigation of a novel S1 binder, can be utilized for identification of other orally available selective serine protease inhibitors.

## Chemistry

**Scheme 1:**
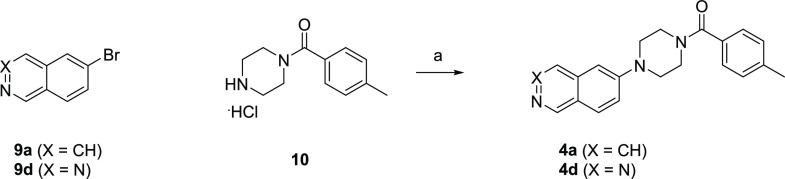
Synthesis of **4a** and **4d** Reagents and conditions: (a) Pd(dba)_2_, DavePhos, NaO^*t*^Bu, toluene, 130 °C, mw, 1 h, 31% (for **4a**) or Pd_2_(dba)_3_, DavePhos, Cs_2_CO_3_, dioxane, 80 °C, 12 h, 25% (for **4d**).

As shown in Scheme 1, **4a** and **4d** were synthesized by the Pd-catalyzed coupling reaction of **9a** and **9d** with **10**, respectively.

**Scheme 2:**
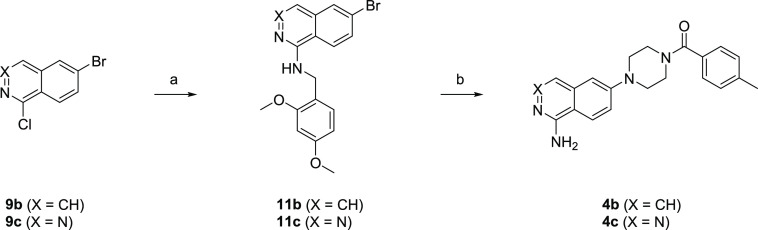
Synthesis of **4b** and **4c** Reagents and conditions: (a) 2,4-dimethoxybenzylamine, 120–130 °C, 3 h to overnight, 52–83%; (b) **10**, Pd(dba)_2_, DavePhos or RuPhos, NaO^*t*^Bu, DME, 100–120 °C, mw, 0.5–1 h, then TFA, rt, 0.5–2 h, 10–11%.

Scheme 2 describes the synthesis of **4b** and **4c**. **9b** and **9c** were treated with 2,4-dimethoxybenzylamine to afford **11b** and **11c** followed by a coupling reaction with **10** and deprotection to give the target compounds **4b** and **4c**, respectively.

**Scheme 3:**
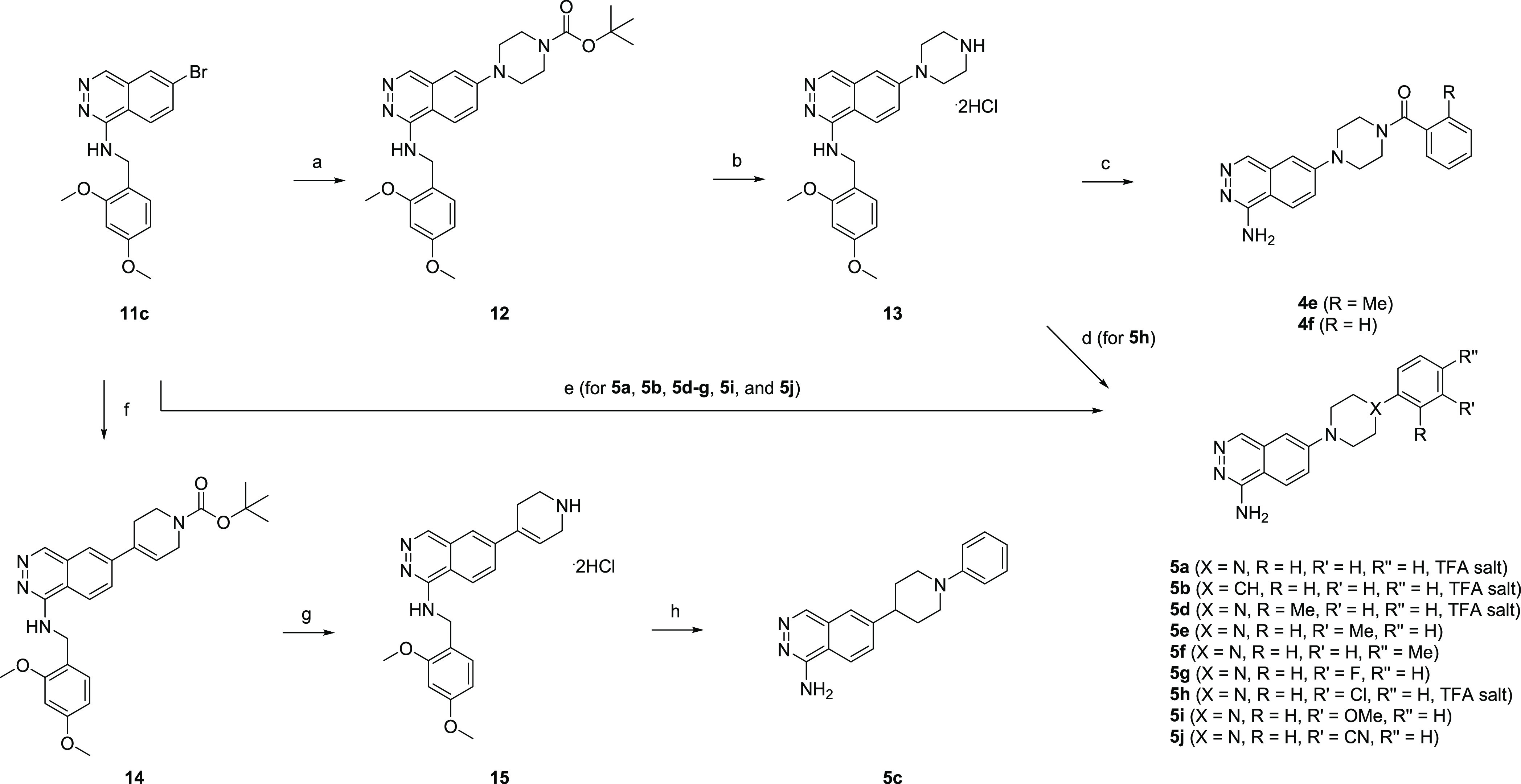
Synthesis of **4e**, **4f**, and **5a**–**j** Reagents and conditions: (a) *tert*-butyl piperazine-1-carboxylate, Pd(dba)_2_, RuPhos, NaO^*t*^Bu, DME, 110 °C, mw, 1 h, 42%; (b) 4 M HCl/EtOAc, MeOH, rt, 2 h, 90%; (c) (substituted)benzoyl chloride, DIPEA, DMF, rt, overnight, then TFA, rt, overnight, 30–32%; (d) 1-chloro-3-iodobenzene, Pd(dba)_2_, RuPhos, NaO^*t*^Bu, DME, 120 °C, mw, 1.5 h, then TFA, rt, overnight, 19%; (e) substituted phenyl piperazine or 4-phenylpiperidine, Pd(dba)_2_, DavePhos or RuPhos, NaO^*t*^Bu, DME, 110–120 °C, mw, 1–3 h, then TFA, rt, 2 h–overnight, 11–21%; (f) *tert*-butyl 4-(4,4,5,5-tetramethyl-1,3,2-dioxaborolan-2-yl)-3,6-dihydropyridine-1(2H)-carboxylate, PdCl_2_(dppf), Cs_2_CO_3_, DME, water, 120 °C, mw, 1 h, 75%; (g) 4 M HCl/EtOAc, MeOH, rt, 2 h, 88%; (h) bromobenzene, Pd(dba)_2_, DavePhos, NaO^*t*^Bu, DME, 120 °C, mw, 1.5 h, then TFA, rt, overnight, then H_2_, Pd/C, MeOH, THF, rt, overnight, 3.4%.

Scheme 3 illustrates the synthesis of **4e**, **4f**, and **5a–j**. **11c** was subjected to a coupling reaction with *tert*-butyl piperazine-1-carboxylate to afford **12**, which was deprotected to give the piperazine dihydrochloride salt **13**. Subsequent acylation and deprotection afforded the target compounds **4e** and **4f**. A coupling reaction of **13** with 1-chloro-3-iodobenzene followed by deprotection gave the target compound **5h**. **11c** was coupled with the corresponding substituted phenyl piperazines or 4-phenyl piperidine followed by deprotection to give the target compounds **5a**, **5b**, **5d–g**, **5i**, and **5j**. A coupling reaction of **11c** with boronic ester gave **14**, which was deprotected to afford tetrahydropyridine **15**. The subsequent coupling reaction, deprotection, and hydrogenation gave the target compound **5c**.

**Scheme 4:**
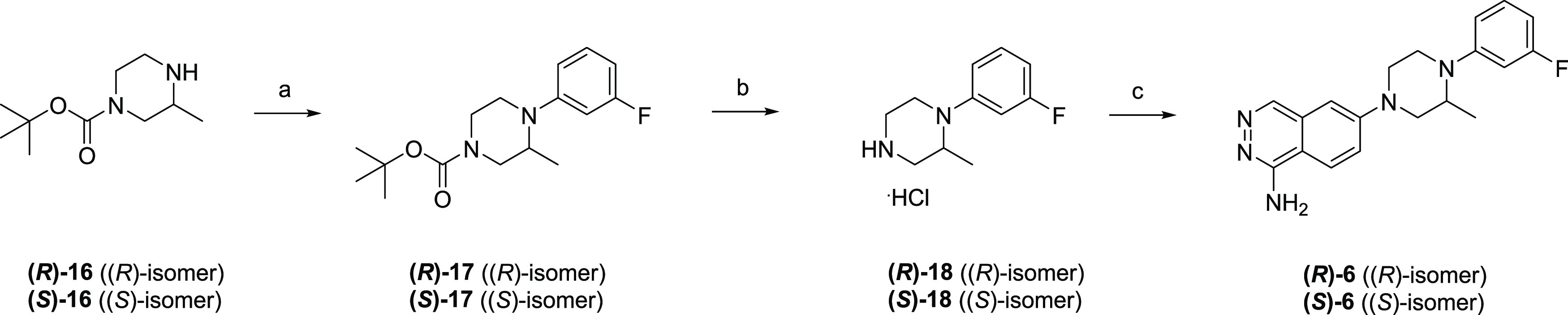
Synthesis of **(*R*)-6** and **(*S*)-6** Reagents and conditions: (a) 1-bromo-3-fluorobenzene, Pd_2_(dba)_3_, *rac*-BINAP, NaO^*t*^Bu, toluene, 110 °C, mw, 1.5 h, 36–42%; (b) 4 M HCl/EtOAc, rt, 3 h, 90–96%; (c) **11c**, Pd(dba)_2_, DavePhos, NaO^*t*^Bu, DME, 110–120 °C, mw, 1.5–2 h, then TFA, rt, 2 h–overnight, 13–21%.

Scheme 4 depicts the synthesis of **(*R*)-6** and **(*S*)-6**. The Boc-protected chiral piperazine **(*R*)-16** and **(*S*)-16** were subjected to a coupling reaction with 1-bromo-3-fluorobenzene to afford **(*R*)-17** and **(*S*)-17**, respectively. Subsequent deprotection gave the precursor hydrochloride salts **(*R*)-18** and **(*S*)-18**, which were coupled with **11c** followed by deprotection to afford the target compounds **(*R*)-6** and **(*S*)-6**, respectively.

**Scheme 5:**
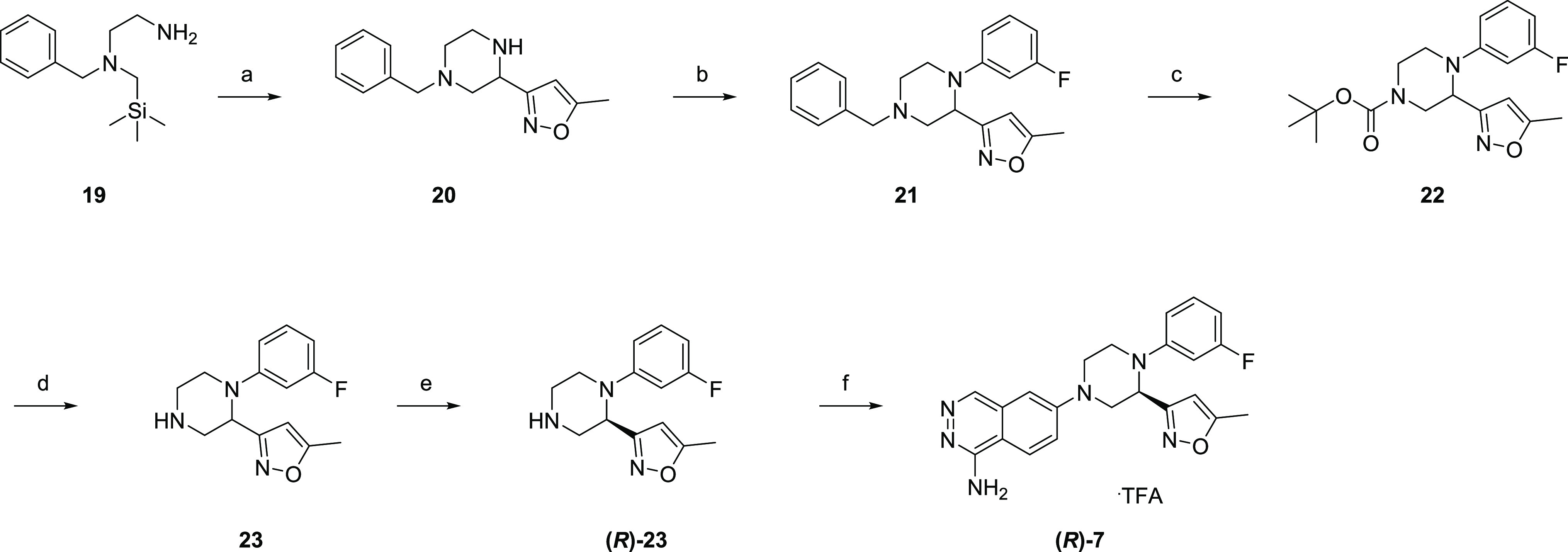
Synthesis of **(*R*)-7** Reagents and conditions: (a) 5-methylisoxazole-3-carbaldehyde, MS4Å, acetonitrile, rt, overnight, then [Ir(ppy)_2_(dtbpy)]PF_6_, acetonitrile/2,2,2-trifluoroethanol, blue LED (30 W), rt, 3 h, 63%; (b) 1-bromo-3-fluorobenzene, Pd(dba)_2_, DavePhos, NaO^*t*^Bu, DME, 110 °C, mw, 2 h, 37%; (c) 1-chloroethyl chloroformate, acetonitrile, rt, 3 h, then MeOH, rt, overnight and reflux, 3 h, then Boc_2_O, Et_3_N, THF, rt, overnight, 92%; (d) 4 M HCl/EtOAc, rt, overnight, 88%; (e) optical resolution, CHIRALPAK IC, 45%; (f) **11c**, Pd(dba)_2_, RuPhos, NaO^*t*^Bu, DME, 120 °C, mw, 40 min, then TFA, rt, 0.5 h, 26%.

Scheme 5 describes the synthesis of **(*R*)-7**. The key intermediate, isoxazole-substituted piperazine **20**, was prepared in a single step from the SLAP reagent **19**.^22^ The reaction worked well in gram-scale synthesis. A coupling reaction of **20** with 1-bromo-3-fluorobenzene afforded **21**, which was converted to Boc-protected piperazine **22**. Subsequent deprotection gave the precursor **23**, which was optically resolved to afford **(*R*)-23**. A subsequent coupling reaction with **11c** and deprotection gave the target compound **(*R*)-7**.

**Scheme 6:**
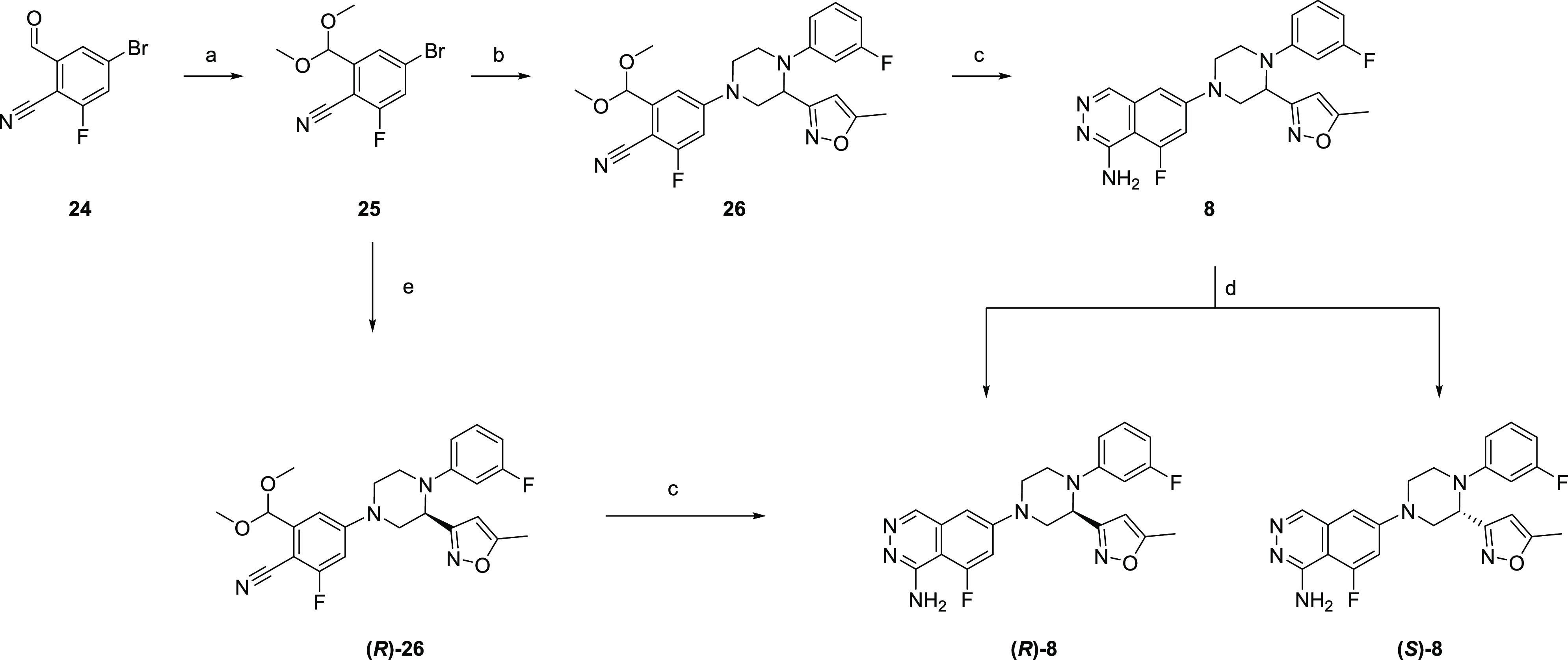
Synthesis of **(*R*)-8** and **(*S*)-8** Reagents and conditions: (a) trimethoxymethane, MeOH, *p*-TsOH, 60 °C, 1 h, 92%; (b) **23**, Pd(dba)_2_, RuPhos, 8 M NaOH aq., DME, 120 °C, mw, 2 h, 72%; (c) 1 M HCl aq., THF, 60 °C, 3–4 h, then hydrazine hydrochloride, DMF, 110 °C, 1.5–3 h, 12–15%; (d) optical resolution, CHIRALCEL OD, 39–42%; (e) **(*R*)-23**, Pd(dba)_2_, RuPhos, NaO^*t*^Bu, DME, 120 °C, mw, 1.5 h, 71%.

Scheme 6 illustrates the synthesis of **(*R*)-8** and **(*S*)-8**. The starting compound **24** was protected to afford **25**, which was subjected to a coupling reaction with **23** to give the precursor **26**. Subsequent phthalazine construction via cyclization gave **8**, which was optically resolved to afford the target compounds **(*R*)-8** and **(*S*)-8**. **(*R*)-8** was also synthesized from **25** and the chiral piperazine **(*R*)-23** via the chiral precursor **(*R*)-26**.

**Figure 11:**
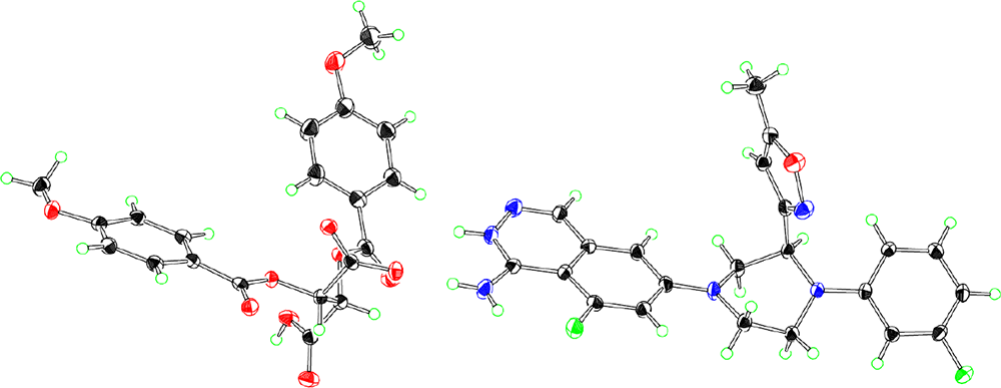
ORTEP of **(*S*)-8**; thermal ellipsoids are drawn at 50% probability. The water molecule is not shown.

The absolute configuration of **(*S*)-8** was confirmed to be the (*S*)-form by single-crystal X-ray structure analysis as a salt with (*S*,*S*)-di-*p*-anisoyltartaric acid, whose ORTEP representation is shown in Figure 11.

## Conclusions

To discover potent, selective, orally available, and brain-penetrable C1s inhibitors, we explored a series of non-amidine compounds based on HTS hit **3**. On the basis of the predicted binding pose of **3**, isoquinoline, the presumed S1 binder, was replaced with 1-aminophthalazine to enhance C1s inhibitory activity by around 10-fold while exhibiting good selectivity against not only C1r, MASP-2, and factor D, serine proteases involved in the complement pathway, but also other serine proteases such as trypsin and thrombin. The crystal structure of a complex of C1s and a small-molecule inhibitor was solved, which demonstrated that the 1-aminophthalazine moiety of **4e** fits tightly in the S1 site and suggested further opportunities to enhance C1s inhibitory activity by structure-based chemical modification around the S2 and S3 site. We successfully enhanced C1s inhibitory activity by acquisition of an additional interaction in the S2 pocket via plausible CH−π interactions with Phe526. Furthermore, in silico-based hot spot prediction aided our investigation into suitable substituents to introduce in the S3 pocket to obtain additional interactions. As a result, the 5-methyl-isoxazole analogue **(*R*)-7**, designed to interact with Gly656 and the nearby wall in the S3 site, further boosted C1s inhibitory activity showing a single-digit nanomolar IC_50_ value. Although membrane permeability of **(*R*)-7** was suboptimal, introduction of a fluorine atom at the 8-position of the 1-aminophthalazine significantly improved the permeability, which led to identification of **(*R*)-8** as a potent, selective, orally available, and brain-penetrable C1s inhibitor. Compound **(*R*)-8** significantly inhibited MAC formation induced by human serum in a dose-dependent manner in the in vitro assay system, proving that selective inhibition of C1s blocked the classical complement pathway effectively. Oral administration of **(*R*)-8** in mice showed reasonable exposure both in plasma and the brain, demonstrating that **(*R*)-8** could work as a valuable tool compound for both in vitro and in vivo assessment by modulating the classical complement pathway.

## Experimental Section

### Chemistry

^1^H NMR and ^13^C NMR spectra were recorded on a Bruker AVANCE III (300 MHz) or a Bruker Advance III plus (400 MHz) spectrometer. Chemical shifts are given in parts per million (ppm) downfield from tetramethylsilane (δ) as the internal standard in a deuterated solvent, and coupling constants (*J*) are in Hertz (Hz). Data are reported as follows: chemical shift, integration, multiplicity (s = singlet, d = doublet, t = triplet, q = quartet, m = multiplet, dd = doublet of doublets, td = triplet of doublets, and br s = broad singlet), and coupling constants. Unless otherwise noted, reagents and solvents were obtained from commercial sources and used without further purification. Thin-layer chromatography (TLC) was performed on silica gel 60F254 plates (Merck) or NH TLC plates (Fuji Silysia Chemical Ltd.). Chromatographic purification was performed on a Purif-Pack (SI or NH, Fuji Silysia Chemical Ltd.) or on a UNIVERSAL column (silica or amino, YAMAZEN Corporation). LC–MS analysis was performed on a Shimadzu liquid chromatography–mass spectrometer system, operating in the APCI (+ or −) or ESI (+ or −) ionization mode. Analytes were eluted using a linear gradient of 0.05% TFA-containing water/acetonitrile or a 5 mM ammonium acetate-containing water/acetonitrile mobile phase and detected at 220 nm. The purities of compounds submitted for biological evaluation were >95% as determined by analytical HPLC unless otherwise noted. Analytical HPLC was performed with a corona charged aerosol detector (CAD), a nanoquantity analyte detector (NQAD), or a photodiode array detector. The column was a Capcell Pak C18AQ (50 mm × 3.0 mm I.D., Shiseido, Japan) or L-column 2 ODS (30 mm × 2.0 mm I.D., CERI, Japan) with a temperature of 50 °C and a flow rate of 0.5 mL/min. Mobile phases A and B under a neutral condition were a mixture of 50 mmol/L ammonium acetate, water, and acetonitrile (1:8:1, v/v/v) and a mixture of 50 mmol/L ammonium acetate and acetonitrile (1:9, v/ v), respectively. The ratio of the mobile phase B was increased linearly from 5 to 95% over 3 min and 95% over the next 1 min. Mobile phases A and B under an acidic condition were a mixture of 0.2% formic acid in 10 mmol/L ammonium formate and 0.2% formic acid in acetonitrile, respectively. The ratio of the mobile phase B was increased linearly from 14% to 86% over 3 min and 86% over the next 1 min. Elemental analyses were performed by Sumika Chemical Analysis Service, and all results were within ±0.4% of the calculated values. Yields are not optimized.

#### [4-(Isoquinolin-6-yl)piperazin-1-yl](4-methylphenyl)methanone (**4a**)

A mixture of **9a** (150 mg, 0.719 mmol), **10** (260 mg, 1.08 mmol), Pd(dba)_2_ (41.5 mg, 0.0719 mmol), DavePhos (14.2 mg, 0.0360 mmol), NaO^*t*^Bu (243 mg, 2.52 mmol), and toluene (2 mL) was stirred at 130 °C for 1 h under microwave irradiation. The precipitate was filtered off, and the filtrate was concentrated in vacuo. The residue was purified by column chromatography (NH silica gel, hexane/EtOAc = 90/10 to 0/100) and preparative HPLC (YMC-Actus Triart C18, eluted with water containing 10 mM NH_4_HCO_3_ in acetonitrile). The desired fractions were concentrated in vacuo, and the residue was washed with EtOAc to give the title compound (74.4 mg, 31%) as a white solid. ^1^H NMR (400 MHz, DMSO-*d*_6_): δ 2.36 (3H, s), 3.35–3.93 (8H, m), 7.14 (1H, s), 7.25–7.31 (2H, m), 7.33–7.39 (2H, m), 7.45–7.57 (2H, m), 7.94 (1H, d, *J* = 9.0 Hz), 8.30 (1H, d, *J* = 5.6 Hz), 9.03 (1H, s). ^13^C NMR (75 MHz, DMSO-*d*_6_): δ 21.4, 48.0, 106.9, 119.7, 119.9, 123.5, 127.6, 129.0, 129.4, 133.3, 137.5, 139.8, 143.6, 151.6, 151.9, 169.7. MS (ESI/APCI) *m*/*z*: 332.2 [M + H]^+^. Anal. calcd for C_21_H_21_N_3_O: C, 76.11; H, 6.39; N, 12.68. Found: C, 76.01; H, 6.43; N, 12.38.

#### [4-(1-Aminoisoquinolin-6-yl)piperazin-1-yl](4-methylphenyl)methanone (**4b**)

A mixture of **11b** (130 mg, 0.348 mmol), **10** (84.0 mg, 0.348 mmol), Pd(dba)_2_ (40.1 mg, 0.0697 mmol), RuPhos (13.0 mg, 0.0279 mmol), NaO^*t*^Bu (100 mg, 1.04 mmol), and DME (2 mL) was stirred at 120 °C for 30 min under microwave irradiation. The precipitate was removed by filtration, and the filtrate was concentrated in vacuo. To the residue was added TFA (2 mL), and the mixture was stirred for 30 min and then concentrated in vacuo. The insoluble material was removed by filtration, and the filtrate was concentrated in vacuo. The residue was purified by preparative HPLC (L-Column 2 ODS, eluted with water in acetonitrile containing 0.1% TFA). The desired fraction was neutralized with sat. NaHCO_3_ aq. and extracted with EtOAc. The organic layer was separated, dried over MgSO_4_, and concentrated in vacuo to give the title compound (12.0 mg, 10%) as an off-white solid. ^1^H NMR (300 MHz, DMSO-*d*_6_): δ 2.36 (3H, s), 3.33–3.41 (4H, m), 3.47–3.86 (4H, m), 6.52 (2H, s), 6.71 (1H, d, *J* = 5.9 Hz), 6.95 (1H, d, *J* = 2.5 Hz), 7.22–7.37 (5H, m), 7.64 (1H, d, *J* = 5.9 Hz), 8.02 (1H, d, *J* = 9.3 Hz). ^13^C NMR (101 MHz, DMSO-*d*_6_): δ 20.9, 47.7, 107.6, 109.5, 111.1, 116.6, 125.2, 127.1, 128.9, 132.8, 138.5, 139.3, 141.9, 151.1, 156.9, 169.2. MS (ESI/APCI) *m*/*z*: 347.2 [M + H]^+^. Anal. calcd for C_21_H_22_N_4_O·0.4H_2_O: C, 71.32; H, 6.50; N, 15.84. Found: C, 71.35; H, 6.46; N, 15.55. Purity 93.4% (analytical HPLC).

#### [4-(1-Aminophthalazin-6-yl)piperazin-1-yl](4-methylphenyl)methanone (**4c**)

A mixture of **11c** (150 mg, 0.401 mmol), **10** (96.0 mg, 0.401 mmol), Pd(dba)_2_ (23.1 mg, 0.0401 mmol), DavePhos (7.89 mg, 0.0200 mmol), NaO^*t*^Bu (96.0 mg, 1.00 mmol), and DME (2 mL) was stirred at 100 °C for 1 h under microwave irradiation. The mixture was poured into water and extracted with EtOAc. The organic layer was separated, washed with water and brine, dried over MgSO_4_, and concentrated in vacuo. To the residue was added TFA (2 mL), and the mixture was stirred for 2 h. The insoluble material was removed by filtration, and the filtrate was concentrated in vacuo. The residue was purified by column chromatography (NH silica gel, EtOAc/MeOH = 100/0 to 80/20) to give the title compound (15.0 mg, 11%) as a white solid. ^1^H NMR (300 MHz, DMSO-*d*_6_): δ 2.36 (3H, s), 3.38–3.82 (8H, m), 6.73 (2H, br s), 7.15 (1H, d, *J* = 2.5 Hz), 7.25–7.30 (2H, m), 7.33–7.39 (2H, m), 7.55–7.62 (1H, m), 8.09 (1H, d, *J* = 9.3 Hz), 8.69 (1H, s). ^13^C NMR (75 MHz, DMSO-*d*_6_): δ 20.9, 47.2, 106.7, 110.2, 120.9, 124.1, 127.2, 128.9, 129.1, 132.8, 139.3, 143.2, 152.2, 155.7, 169.2. MS (ESI/APCI) *m*/*z*: 348.2 [M + H]^+^. Anal. calcd for C_20_H_21_N_5_O·0.2H_2_O: C, 68.43; H, 6.14; N, 19.95. Found: C, 68.14; H, 6.23; N, 19.91.

#### (4-Methylphenyl)[4-(phthalazin-6-yl)piperazin-1-yl]methanone (**4d**)

A mixture of **9d** (100 mg, 0.478 mmol), **10** (173 mg, 0.718 mmol), Pd_2_(dba)_3_ (87.6 mg, 0.0957 mmol), DavePhos (37.7 mg, 0.0957 mmol), Cs_2_CO_3_ (311 mg, 0.957 mmol), and dioxane (3 mL) was stirred at 80 °C for 12 h under a N_2_ atmosphere. The mixture was diluted with water and extracted with EtOAc. The combined organic layer was washed with brine, dried over anhydrous Na_2_SO_4_, and filtered. The filtrate was concentrated in vacuo. The residue was purified by preparative TLC (silica gel plate, EtOAc) and preparative HPLC (Phenomenex Gemini, eluted with water containing 0.04% NH_3_H_2_O and 10 mM NH_4_HCO_3_ in acetonitrile) to give the title compound (82 mg, 25%) as a yellow solid. ^1^H NMR (400 MHz, DMSO-*d*_6_): δ 2.37 (3H, s), 3.42–3.87 (8H, m), 7.27–7.31 (2H, m), 7.33 (1H, d, *J* = 2.4 Hz), 7.36–7.40 (2H, m), 7.79 (1H, dd, *J* = 8.8, 2.4 Hz), 7.98 (1H, d, *J* = 9.2 Hz), 9.35 (1H, d, *J* = 1.2 Hz), 9.41 (1H, s). ^13^C NMR (75 MHz, CDCl_3_): δ 21.4, 48.2, 77.2, 106.8, 120.9, 123.2, 127.3, 127.7, 128.5, 129.2, 132.2, 140.4, 149.9, 150.6, 153.2, 170.8. MS (ESI/APCI) *m*/*z*: 333.2 [M + H]^+^. Anal. calcd for C_20_H_20_N_4_O·0.2H_2_O: C, 71.49; H, 6.12; N, 16.67. Found: C, 71.34; H, 6.17; N, 16.51.

#### [4-(1-Aminophthalazin-6-yl)piperazin-1-yl](2-methylphenyl)methanone (**4e**)

A mixture of **13** (80.0 mg, 0.177 mmol), DIPEA (0.124 mL, 0.707 mmol), 2-methylbenzoyl chloride (0.0230 mL, 0.177 mmol), and DMF (2 mL) was stirred at room temperature overnight. The mixture was poured into water and extracted with EtOAc. The organic layer was separated, washed with water and brine, dried over MgSO_4_, and concentrated in vacuo. To the residue, TFA (1 mL) was added, and the mixture was stirred at room temperature overnight. The mixture was concentrated in vacuo. The mixture was purified by column chromatography (NH silica gel, hexane/EtOAc = 50/50 to 0/100 and then EtOAc/MeOH = 100/0 to 70/30) and preparative HPLC (YMC-Actus Triart C18, eluted with water containing 10 mM NH_4_HCO_3_ in acetonitrile). The desired fraction was concentrated in vacuo to give the title compound (18.1 mg, 30%) as a white solid. ^1^H NMR (400 MHz, DMSO-*d*_6_): δ 2.25 (3H, s), 3.26–3.32 (4H, m), 3.42–3.58 (2H, m), 3.75–3.95 (2H, m), 6.69 (2H, s), 7.15 (1H, d, *J* = 1.5 Hz), 7.20–7.39 (4H, m), 7.58 (1H, dd, *J* = 9.1, 1.7 Hz), 8.08 (1H, d, *J* = 9.0 Hz), 8.69 (1H, s). ^13^C NMR (75 MHz, DMSO-*d*_6_): δ 19.1, 41.0, 46.2, 47.7, 48.0, 107.3, 110.7, 121.5, 124.6, 126.2, 126.3, 129.2, 129.6, 130.7, 134.3, 136.6, 143.6, 152.7, 156.2, 169.1. MS (ESI/APCI) *m*/*z*: 348.0 [M + H]^+^. Anal. calcd for C_20_H_21_N_5_O·0.1H_2_O: C, 68.79; H, 6.12; N, 20.05. Found: C, 68.77; H, 6.20; N, 19.87.

#### [4-(1-Aminophthalazin-6-yl)piperazin-1-yl](phenyl)methanone (**4f**)

The title compound was prepared in 32% yield using benzoyl chloride in an analogous manner to **4e** as a pale yellow solid. ^1^H NMR (300 MHz, DMSO-*d*_6_): δ 3.35–4.00 (8H, m), 6.70 (2H, s), 7.15 (1H, d, *J* = 2.3 Hz), 7.41–7.51 (5H, m), 7.58 (1H, dd, *J* = 9.0, 2.6 Hz), 8.08 (1H, d, *J* = 9.0 Hz), 8.69 (1H, s). ^13^C NMR (75 MHz, DMSO-*d*_6_): δ 47.7, 107.2, 110.7, 121.4, 124.6, 127.5, 128.9, 129.6, 130.1, 136.2, 143.7, 152.7, 156.2, 169.6. MS (ESI/APCI) *m*/*z*: 334.2 [M + H]^+^. Anal. calcd for C_19_H_19_N_5_O·0.1H_2_O: C, 68.08; H, 5.77; N, 20.89. Found: C, 67.97; H, 5.87; N, 20.74.

#### 6-(4-Phenylpiperazin-1-yl)phthalazin-1-amine Trifluoroacetic Acid Salt (**5a**)

A mixture of **11c** (300 mg, 0.802 mmol), 1-phenylpiperazine (260 mg, 1.60 mmol), Pd(dba)_2_ (46.1 mg, 0.0801 mmol), RuPhos (37.4 mg, 0.0801 mmol), NaO^*t*^Bu (154 mg, 1.60 mmol), and DME (5 mL) was stirred at 120 °C under microwave irradiation for 1 h. The precipitate was filtered off, and the filtrate was concentrated in vacuo. To the residue was added TFA (3 mL). The mixture was stirred at room temperature for 2 h. The mixture was concentrated in vacuo. The residue was purified by column chromatography (NH silica gel, hexane/EtOAc = 50/50 to 0/100 and then EtOAc/MeOH = 100/0 to 80/20) and preparative HPLC (YMC-Actus Triart C18, eluted with water in acetonitrile containing 0.1% TFA). The desired fraction was concentrated in vacuo to give the title compound (60.6 mg, 18%) as a white solid. ^1^H NMR (400 MHz, DMSO-*d*_6_): δ 3.30–3.38 (4H, m), 3.68–3.77 (4H, m), 6.83 (1H, t, *J* = 7.2 Hz), 7.02 (2H, d, *J* = 8.2 Hz), 7.22–7.30 (2H, m), 7.49 (1H, d, *J* = 1.7 Hz), 7.76–7.85 (1H, m), 8.43 (1H, d, *J* = 9.4 Hz), 8.65 (1H, s), 8.77 (2H, br s), 13.87 (1H, br s). ^13^C NMR (101 MHz, DMSO-*d*_6_): δ 46.5, 48.2, 108.3, 109.0, 117.5 (q, *J* = 298.6 Hz), 116.1, 119.7, 120.9, 127.0, 129.5, 130.8, 143.8, 151.0, 152.6, 155.1, 159.5 (q, *J* = 31.5 Hz). MS (ESI/APCI) *m*/*z*: 306.2 [M + H-TFA]^+^. Anal. calcd for C_20_H_20_F_3_N_5_O_2_·0.1H_2_O: C, 57.03; H, 4.83; N, 16.63. Found: C, 56.77; H, 4.94; N, 16.48.

#### 6-(4-Phenylpiperidin-1-yl)phthalazin-1-amine Trifluoroacetic Acid Salt (**5b**)

A mixture of **11c** (57.0 mg, 0.152 mmol), 4-phenylpiperidine (24.6 mg, 0.152 mmol), Pd(dba)_2_ (8.76 mg, 0.0152 mmol), DavePhos (3.00 mg, 0.00762 mmol), NaO^*t*^Bu (29.3 mg, 0.305 mmol), and DME (2 mL) was stirred at 110 °C under microwave irradiation for 1 h. The mixture was poured into water and extracted with EtOAc. The organic layer was separated, washed with water and brine, dried over MgSO_4_, and concentrated in vacuo. To the residue was added TFA (1 mL), and the mixture was stirred at room temperature overnight. The mixture was concentrated in vacuo. The residue was purified by preparative HPLC (L-Column 2 ODS, eluted with water in acetonitrile containing 0.1% TFA). The desired fraction was concentrated in vacuo to give the title compound (7.20 mg, 11%) as a yellow solid. ^1^H NMR (300 MHz, DMSO-*d*_6_): δ 1.59–1.82 (2H, m), 1.87–1.99 (2H, m), 2.82–3.00 (1H, m), 3.09–3.25 (2H, m), 4.20–4.42 (2H, m), 7.13–7.36 (5H, m), 7.48 (1H, d, *J* = 2.6 Hz), 7.78 (1H, dd, *J* = 9.4, 2.6 Hz), 8.40 (1H, d, *J* = 9.0 Hz), 8.62 (1H, s), 8.82 (2H, br s), 13.99 (1H, br s). ^13^C NMR (101 MHz, DMSO-*d*_6_): δ 32.2, 41.5, 47.1, 107.7, 107.8, 117.2 (q, *J* = 299.8 Hz), 120.4, 126.2, 126.6, 126.7, 128.4, 130.5, 143.4, 145.4, 151.8, 154.5, 158.4 (q, *J* = 30.8 Hz). MS (ESI/APCI) *m*/*z*: 305.2 [M + H-TFA]^+^. Anal. calcd for C_21_H_21_F_3_N_4_O_2_·0.3H_2_O: C, 59.51; H, 5.14; N, 13.22. Found: C, 59.23; H, 5.19; N, 13.31.

#### 6-(1-Phenylpiperidin-4-yl)phthalazin-1-amine (**5c**)

A mixture of **15** (478 mg, 1.06 mmol), bromobenzene (251 mg, 1.60 mmol), Pd(dba)_2_ (122 mg, 0.212 mmol), DavePhos (84.0 mg, 0.212 mmol), NaO^*t*^Bu (409 mg, 4.25 mmol), and DME (6 mL) was stirred at 120 °C under microwave irradiation for 1.5 h. The precipitate was filtered off, and the filtrate was concentrated in vacuo. To the residue was added TFA (2 mL). The mixture was stirred at room temperature overnight. The mixture was purified by column chromatography (NH silica gel, hexane/EtOAc = 50/50 to 0/100 and then EtOAc/MeOH = 100/0 to 80/20) to give 6-(1-phenyl-1,2,3,6-tetrahydropyridin-4-yl)phthalazin-1-amine (61.8 mg, including impurities), which was combined with Pd/C (10% on carbon, wetted with ca. 50% water, 30 mg), MeOH (3 mL), and THF (0.5 mL). Under a H_2_ atmosphere, the mixture was stirred at room temperature overnight. The catalyst was filtered off, and the filtrate was concentrated in vacuo. The mixture was purified by column chromatography (NH silica gel, hexane/EtOAc = 50/50 to 0/100 and then EtOAc/MeOH = 100/0 to 80/20) and preparative HPLC (YMC-Actus Triart C18, eluted with water containing 10 mM NH_4_HCO_3_ in acetonitrile). The desired fraction was concentrated in vacuo to give the title compound (10.9 mg, 3.4%) as a colorless solid. ^1^H NMR (300 MHz, DMSO-*d*_6_): δ 1.74–2.03 (4H, m), 2.71–2.99 (3H, m), 3.77–3.93 (2H, m), 6.73–6.82 (1H, m), 6.89 (2H, s), 7.00 (2H, d, *J* = 7.9 Hz), 7.17–7.31 (2H, m), 7.72–7.87 (2H, m), 8.19 (1H, d, *J* = 8.3 Hz), 8.85 (1H, s). ^13^C NMR (101 MHz, DMSO-*d*_6_): δ 32.3, 41.7, 49.2, 115.8, 115.9, 118.6, 122.9, 122.9, 127.5, 128.9, 130.8, 143.3, 149.9, 151.2, 156.0. MS (ESI/APCI) *m*/*z*: 305.2 [M + H]^+^. Anal. calcd for C_19_H_20_N_4_·0.1H_2_O: C, 74.53; H, 6.65; N, 18.30. Found: C, 74.55; H, 6.62; N, 18.31.

#### 6-[4-(2-Methylphenyl)piperazin-1-yl]phthalazin-1-amine Trifluoroacetic Acid Salt (**5d**)

The title compound was prepared in 11% yield using 1-(2-methylphenyl)piperazine in an analogous manner to **5b** as a yellow solid. ^1^H NMR (400 MHz, DMSO-*d*_6_): δ 2.32 (3H, s), 2.88–3.07 (4H, m), 3.59–3.79 (4H, m), 6.97–7.04 (1H, m), 7.07 (1H, d, *J* = 7.9 Hz), 7.14–7.25 (2H, m), 7.50 (1H, s), 7.75–7.85 (1H, m), 8.44 (1H, d, *J* = 9.3 Hz), 8.55–8.93 (3H, m), 13.85 (1H, br s). ^13^C NMR (101 MHz, DMSO-*d*_6_): δ 17.6, 46.8, 51.0, 108.0, 108.6, 117.0 (q, *J* = 299.3 Hz), 118.9, 120.5, 123.3, 126.5, 126.6, 130.2, 130.9, 131.9, 143.4, 150.7, 152.1, 154.9, 159.0 (q, *J* = 31.5 Hz). MS (ESI/APCI) *m*/*z*: 320.3 [M + H-TFA]^+^. Anal. calcd for C_21_H_22_F_3_N_5_O_2_: C, 58.19; H, 5.12; N, 16.16. Found: C, 57.95; H, 5.26; N, 16.14.

#### 6-[4-(3-Methylphenyl)piperazin-1-yl]phthalazin-1-amine (**5e**)

The title compound was prepared in 21% yield using 1-(3-methylphenyl)piperazine in an analogous manner to **5b** as a yellow solid. ^1^H NMR (300 MHz, DMSO-*d*_6_): δ 2.27 (3H, s), 3.26–3.34 (4H, m), 3.46–3.57 (4H, m), 6.60–6.73 (3H, m), 6.78–6.88 (2H, m), 7.13 (1H, t, *J* = 7.7 Hz), 7.19 (1H, d, *J* = 2.3 Hz), 7.64 (1H, dd, *J* = 9.2, 2.4 Hz), 8.09 (1H, d, *J* = 9.4 Hz), 8.72 (1H, s). ^13^C NMR (75 MHz, DMSO-*d*_6_): δ 21.9, 47.7, 48.6, 107.0, 110.6, 113.4, 116.8, 120.6, 121.3, 124.5, 129.3, 129.7, 138.5, 143.7, 151.3, 152.9, 156.2. MS (ESI/APCI) *m*/*z*: 320.3 [M + H]^+^. Anal. calcd for C_19_H_21_N_5_: C, 71.45; H, 6.63; N, 21.93. Found: C, 71.49; H, 6.67; N, 21.85.

#### 6-[4-(4-Methylphenyl)piperazin-1-yl]phthalazin-1-amine (**5f**)

The title compound was prepared in 13% yield using 1-(4-methylphenyl)piperazine in an analogous manner to **5b** as an off-white solid. ^1^H NMR (300 MHz, DMSO-*d*_6_): δ 2.22 (3H, s), 3.20–3.28 (4H, m), 3.41–3.58 (4H, m), 6.67 (2H, s), 6.92 (2H, d, *J* = 8.7 Hz), 7.07 (2H, d, *J* = 8.2 Hz), 7.19 (1H, d, *J* = 2.5 Hz), 7.64 (1H, dd, *J* = 9.2, 2.5 Hz), 8.08 (1H, d, *J* = 9.1 Hz), 8.71 (1H, s). ^13^C NMR (75 MHz, DMSO-*d*_6_): δ 20.5, 47.7, 49.1, 107.0, 110.6, 116.5, 121.3, 124.5, 128.6, 129.7, 129.9, 143.7, 149.2, 153.0, 156.2. MS (ESI/APCI) *m*/*z*: 320.2 [M + H]^+^. Anal. calcd for C_19_H_21_N_5_: C, 71.45; H, 6.63; N, 21.93. Found: C, 71.22; H, 6.80; N, 21.75.

#### 6-[4-(3-Fluorophenyl)piperazin-1-yl]phthalazin-1-amine (**5g**)

A mixture of **11c** (80.0 mg, 0.214 mmol), 1-(3-fluorophenyl)piperazine (77.0 mg, 0.428 mmol), Pd(dba)_2_ (12.3 mg, 0.0214 mmol), DavePhos (16.8 mg, 0.0428 mmol), NaO^*t*^Bu (30.8 mg, 0.321 mmol), and DME (1.5 mL) was stirred at 120 °C under microwave irradiation for 1 h. The precipitate was filtered off, and the filtrate was concentrated in vacuo. To the residue was added TFA (1 mL). The mixture was stirred at room temperature for 2 h. The precipitate was filtered off, and the filtrate was concentrated in vacuo. The residue was purified by preparative HPLC (YMC-Actus Triart C18, eluted with water containing 10 mM NH_4_HCO_3_ in acetonitrile). The desired fraction was concentrated in vacuo to give the title compound (14.8 mg, 21%) as a light brown solid. ^1^H NMR (300 MHz, DMSO-*d*_6_): δ 3.34–3.44 (4H, m), 3.48–3.58 (4H, m), 6.53–6.64 (1H, m), 6.71 (2H, s), 6.78–6.84 (1H, m), 6.84–6.88 (1H, m), 7.20 (1H, d, *J* = 2.3 Hz), 7.21–7.33 (1H, m), 7.64 (1H, dd, *J* = 9.2, 2.4 Hz), 8.09 (1H, d, *J* = 9.4 Hz), 8.71 (1H, s). ^13^C NMR (75 MHz, DMSO-*d*_6_): δ 47.4, 47.9, 102.5 (d, *J* = 24.5 Hz), 105.5 (d, *J* = 21.0 Hz), 107.0, 110.6, 111.5 (d, *J* = 1.7 Hz), 121.3, 124.6, 129.6, 130.9 (d, *J* = 10.0 Hz), 143.7, 152.8, 153.0 (d, *J* = 10.0 Hz), 156.2, 163.8 (d, *J* = 240.5 Hz). MS (ESI/APCI) *m*/*z*: 324.2 [M + H]^+^. Anal. calcd for C_18_H_18_FN_5_·0.1H_2_O: C, 66.49; H, 5.64; N, 21.54. Found: C, 66.61; H, 5.70; N, 21.28.

#### 6-[4-(3-Chlorophenyl)piperazin-1-yl]phthalazin-1-amine Trifluoroacetic Acid Salt (**5h**)

A mixture of **13** (100 mg, 0.221 mmol), 1-chloro-3-iodobenzene (79.0 mg, 0.332 mmol), Pd(dba)_2_ (25.4 mg, 0.0442 mmol), RuPhos (20.6 mg, 0.0442 mmol), NaO^*t*^Bu (85.0 mg, 0.884 mmol), and DME (2 mL) was stirred at 120 °C under microwave irradiation for 1.5 h. The precipitate was filtered off, and the filtrate was concentrated in vacuo. To the residue was added TFA (1 mL). The mixture was stirred at room temperature overnight. The mixture was purified by column chromatography (NH silica gel, hexane/EtOAc = 50/50 to 0/100 and then EtOAc/MeOH = 100/0 to 80/20) and preparative HPLC (YMC-Actus Triart C18, eluted with water in acetonitrile containing 0.1% TFA). The desired fraction was concentrated in vacuo and washed with acetonitrile/MeOH to give the title compound (18.7 mg, 19%) as a white solid. ^1^H NMR (400 MHz, DMSO-*d*_6_): δ 3.36–3.48 (4H, m), 3.64–3.78 (4H, m), 6.83 (1H, d, *J* = 7.8 Hz), 6.94–7.00 (1H, m), 7.03 (1H, s), 7.25 (1H, t, *J* = 8.1 Hz), 7.48 (1H, d, *J* = 1.8 Hz), 7.79 (1H, dd, *J* = 9.4, 1.7 Hz), 8.43 (1H, d, *J* = 9.3 Hz), 8.65 (1H, s), 8.83 (2H, br s), 13.99 (1H, br s). ^13^C NMR (101 MHz, DMSO-*d*_6_): δ 45.8, 46.9, 107.8, 108.6, 117.1 (q, *J* = 299.1 Hz), 113.6, 114.6, 118.3, 120.4, 126.5, 130.2, 130.5, 133.9, 143.3, 151.7, 152.1, 154.5, 159.0 (q, *J* = 31.8 Hz). MS (ESI/APCI) *m*/*z*: 340.1 [M + H-TFA]^+^. Anal. calcd for C_20_H_19_ClF_3_N_5_O_2_: C, 52.93; H, 4.22; N, 15.43. Found: C, 52.96; H, 4.35; N, 15.30. Purity 91.5% (analytical HPLC).

#### 6-[4-(3-Methoxyphenyl)piperazin-1-yl]phthalazin-1-amine (**5i**)

The title compound was prepared in 15% yield using 1-(3-methoxyphenyl)piperazine in an analogous manner to **5g** as a colorless solid. ^1^H NMR (300 MHz, DMSO-*d*_6_): δ 3.25–3.37 (4H, m), 3.51 (4H, dd, *J* = 6.3, 3.8 Hz), 3.74 (3H, s), 6.41 (1H, dd, *J* = 7.9, 2.1 Hz), 6.53 (1H, t, *J* = 2.3 Hz), 6.61 (1H, dd, *J* = 8.2, 1.8 Hz), 6.66 (2H, s), 7.06–7.24 (2H, m), 7.63 (1H, dd, *J* = 9.2, 2.5 Hz), 8.08 (1H, d, *J* = 9.3 Hz), 8.71 (1H, s). ^13^C NMR (75 MHz, DMSO-*d*_6_): δ 47.6, 48.5, 55.4, 102.3, 105.0, 107.0, 108.8, 110.6, 121.3, 124.6, 129.6, 130.2, 143.7, 152.6, 152.9, 156.2, 160.7. MS (ESI/APCI) *m*/*z*: 336.2 [M + H]^+^. Anal. calcd for C_19_H_21_N_5_O: C, 68.04; H, 6.31; N, 20.88. Found: C, 67.83; H, 6.42; N, 20.76.

#### 3-[4-(1-Aminophthalazin-6-yl)piperazin-1-yl]benzonitrile (**5j**)

The title compound was prepared in 18% yield using 3-(piperazin-1-yl)benzonitrile hydrochloride in an analogous manner to **5b** as a white solid. ^1^H NMR (300 MHz, DMSO-*d*_6_): δ 3.41–3.46 (4H, m), 3.50–3.57 (4H, m), 6.67 (2H, s), 7.17–7.23 (2H, m), 7.33–7.47 (3H, m), 7.64 (1H, dd, *J* = 9.2, 2.5 Hz), 8.09 (1H, d, *J* = 9.2 Hz), 8.72 (1H, s). ^13^C NMR (75 MHz, DMSO-*d*_6_): δ 46.8, 47.1, 106.5, 110.1, 112.0, 117.8, 119.3, 119.8, 120.8, 121.9, 124.1, 129.2, 130.2, 143.2, 150.8, 152.3, 155.7. MS (ESI/APCI) *m*/*z*: 331.1 [M + H]^+^. Anal. calcd for C_19_H_18_N_6_·0.1H_2_O: C, 68.70; H, 5.52; N, 25.30. Found: C, 68.49; H, 5.65; N, 25.18.

#### 6-[(3*R*)-4-(3-Fluorophenyl)-3-methylpiperazin-1-yl]phthalazin-1-amine (**(*R*)-6**)

The title compound was prepared in 13% yield using **(*R*)-18** in an analogous manner to **5g** as a white solid. ^1^H NMR (300 MHz, DMSO-*d*_6_): δ 1.09 (3H, d, *J* = 6.4 Hz), 3.01–3.32 (3H, m), 3.49–3.59 (1H, m), 3.81–4.01 (2H, m), 4.12–4.25 (1H, m), 6.49–6.57 (1H, m), 6.68 (2H, s), 6.72–6.81 (2H, m), 7.16 (1H, d, *J* = 2.5 Hz), 7.19–7.29 (1H, m), 7.61 (1H, dd, *J* = 9.2, 2.5 Hz), 8.09 (1H, d, *J* = 9.2 Hz), 8.72 (1H, s). ^13^C NMR (75 MHz, DMSO-*d*_6_): δ 12.9, 41.4, 46.7, 50.0, 51.9, 101.4 (d, *J* = 25.4 Hz), 104.1 (d, *J* = 21.6 Hz), 105.9, 109.8, 110.5 (d, *J* = 2.2 Hz), 120.5, 124.1, 129.2, 130.5 (d, *J* = 10.5 Hz), 143.2, 151.3 (d, *J* = 10.5 Hz), 152.7, 155.7, 163.5 (d, *J* = 239.9 Hz). MS (ESI/APCI) *m*/*z*: 338.2 [M + H]^+^. Anal. calcd for C_19_H_20_FN_5_: C, 67.64; H, 5.97; N, 20.76. Found: C, 67.76; H, 6.09; N, 20.71.

#### 6-[(3*S*)-4-(3-Fluorophenyl)-3-methylpiperazin-1-yl]phthalazin-1-amine (**(*S*)-6**)

The title compound was prepared in 21% yield using **(*S*)-18** in an analogous manner to **5b** as a white solid. ^1^H NMR (300 MHz, DMSO-*d*_6_): δ 1.09 (3H, d, *J* = 6.4 Hz), 3.02–3.32 (3H, m), 3.49–3.58 (1H, m), 3.80–4.03 (2H, m), 4.12–4.24 (1H, m), 6.46–6.60 (1H, m), 6.70 (2H, s), 6.72–6.82 (2H, m), 7.16 (1H, d, *J* = 2.5 Hz), 7.19–7.30 (1H, m), 7.61 (1H, dd, *J* = 9.2, 2.5 Hz), 8.10 (1H, d, *J* = 9.2 Hz), 8.72 (1H, s). ^13^C NMR (75 MHz, DMSO-*d*_6_): δ 12.9, 41.4, 46.7, 50.0, 51.9, 101.4 (d, *J* = 24.5 Hz), 104.1 (d, *J* = 21.6 Hz), 105.9, 109.8, 110.5 (d, *J* = 1.7 Hz), 120.5, 124.1, 129.2, 130.5 (d, *J* = 10.0 Hz), 143.2, 151.3 (d, *J* = 10.5 Hz), 152.7, 155.7, 163.5 (d, *J* = 239.9 Hz). MS (ESI/APCI) *m*/*z*: 338.2 [M + H]^+^. C_19_H_20_FN_5_: C, 67.64; H, 5.97; N, 20.76. Found: C, 67.73; H, 6.04; N, 20.72.

#### 6-[(3*R*)-4-(3-Fluorophenyl)-3-(5-methyl-1,2-oxazol-3-yl)piperazin-1-yl]phthalazin-1-amine Trifluoroacetic Acid Salt (**(*R*)-7**)

The title compound was prepared in 26% yield using **(*R*)-23** in an analogous manner to **5a** as an off-white solid. ^1^H NMR (300 MHz, DMSO-*d*_6_): δ 2.27 (3H, s), 3.40–3.55 (2H, m), 3.69–3.86 (2H, m), 3.93–4.10 (1H, m), 4.37–4.48 (1H, m), 5.32–5.43 (1H, m), 6.08 (1H, d, *J* = 0.8 Hz), 6.44–6.65 (1H, m), 6.76 (2H, s), 7.15–7.30 (1H, m), 7.38 (1H, d, *J* = 2.4 Hz), 7.68 (1H, dd, *J* = 9.3, 2.5 Hz), 8.37 (1H, d, *J* = 9.2 Hz), 8.50–8.70 (3H, m), 13.57–14.02 (1H, m). MS (ESI/APCI) *m*/*z*: 405.2 [M + H-TFA]^+^.

#### 8-Fluoro-6-[4-(3-fluorophenyl)-3-(5-methyl-1,2-oxazol-3-yl)piperazin-1-yl]phthalazin-1-amine (**8**)

To a solution of **26** (1.85 g, 4.07 mmol) in THF (20 mL) was added 1 M HCl aq. (20 mL) at room temperature. The mixture was stirred at 60 °C for 4 h. The mixture was neutralized with sat. NaHCO_3_ aq. at 0 °C and extracted with EtOAc. The organic layer was separated, washed with water and brine, dried over MgSO_4_, and concentrated in vacuo. To the residue in DMF (4 mL) was added hydrazine hydrochloride (279 mg, 4.07 mmol) at room temperature. The mixture was stirred at 110 °C for 3 h. The mixture was concentrated in vacuo. The residue was purified by column chromatography (NH silica gel, EtOAc/MeOH = 100/0 to 85/15) and by additional column chromatography (silica gel, EtOAc/MeOH = 100/0 to 60/40) to give the title compound (200 mg, 12%) as a pale orange solid. ^1^H NMR (300 MHz, DMSO-*d*_6_): δ 2.28 (3H, d, *J* = 0.8 Hz), 3.23–3.30 (1H, m), 3.38–3.49 (1H, m), 3.62 (1H, dd, *J* = 13.0, 3.8 Hz), 3.70–3.83 (1H, m), 3.88–4.00 (1H, m), 4.25–4.35 (1H, m), 5.35 (1H, br s), 6.10 (1H, d, *J* = 0.9 Hz), 6.54 (1H, td, *J* = 7.9, 1.7 Hz), 6.67 (2H, br s), 6.78 (1H, d, *J* = 1.3 Hz), 6.81 (1H, dd, *J* = 5.1, 1.9 Hz), 7.04 (1H, d, *J* = 2.3 Hz), 7.16–7.30 (1H, m), 7.38 (1H, dd, *J* = 16.0, 2.3 Hz), 8.68 (1 h, d, *J* = 3.4 Hz). MS (ESI/APCI) *m*/*z*: 423.2 [M + H]^+^.

#### 8-Fluoro-6-[(3*R*)-4-(3-fluorophenyl)-3-(5-methyl-1,2-oxazol-3-yl)piperazin-1-yl]phthalazin-1-amine (**(*R*)-8**) and 8-Fluoro-6-[(3*S*)-4-(3-fluorophenyl)-3-(5-methyl-1,2-oxazol-3-yl)piperazin-1-yl]phthalazin-1-amine (**(*S*)-8**)

**8** (285 mg, 0.676 mmol) was optically resolved by chiral preparative HPLC (CHIRALCEL OD, 50 mm I.D. × 500 mm *L*, 20 μm with MeOH/diethylamine = 1000/1(v/v)). The first eluting fraction was collected and concentrated in vacuo to give **(*S*)-8** (111 mg, 39%) as a white solid. ^1^H NMR (300 MHz, DMSO-*d*_6_): δ 2.28 (3H, d, *J* = 0.8 Hz), 3.20–3.31 (1H, m), 3.37–3.50 (1H, m), 3.59 (1H, dd, *J* = 13.0, 3.8 Hz), 3.70–3.82 (1H, m), 3.95 (1H, d, *J* = 12.7 Hz), 4.27 (1H, d, *J* = 12.4 Hz), 5.34 (1H, br s), 6.11 (1H, d, *J* = 0.9 Hz), 6.46 (2H, s), 6.50–6.65 (1H, m), 6.78 (1H, s), 6.79–6.87 (1H, m), 7.01 (1H, d, *J* = 2.3 Hz), 7.16–7.30 (1H, m), 7.36 (1H, dd, *J* = 15.9, 2.3 Hz), 8.69 (1H, d, *J* = 3.5 Hz). ^13^C NMR (75 MHz, DMSO-*d*_6_): δ 11.7, 42.6, 46.1, 49.5, 51.6, 99.2 (d, *J* = 13.3 Hz), 101.2, 101.2 (d, *J* = 25.4 Hz), 102.2 (d, *J* = 2.2 Hz), 104.5 (d, *J* = 21.0 Hz), 105.0 (d, *J* = 26.5 Hz), 110.1 (d, *J* = 1.7 Hz), 130.4 (d, *J* = 10.0 Hz), 130.6 (d, *J* = 4.4 Hz), 142.3 (d, *J* = 2.8 Hz), 150.9 (d, *J* = 10.0 Hz), 152.7 (d, *J* = 9.4 Hz), 152.8 (d, *J* = 1.7 Hz), 158.8 (d, *J* = 249.9 Hz), 162.7, 163.3 (d, *J* = 240.5 Hz), 169.4. MS (ESI/APCI) *m*/*z*: 423.2 [M + H]^+^. Anal. calcd for C_22_H_20_F_2_N_6_O·0.3H_2_O: C, 61.76; H, 4.85; N, 19.64. Found: C, 62.12; H, 4.86; N, 19.27. Chiral HPLC analysis (CHIRALCEL OD-H 4.6 mm I.D. × 250 mm *L*, 5 μm with MeOH/diethylamine = 1000/1(v/v)): *t*_R_ = 7.84 min and >99% ee. The second eluting fraction was collected and concentrated in vacuo to give **(*R*)-8** (119 mg, 42%) as a white solid. ^1^H NMR (300 MHz, DMSO-*d*_6_): δ 2.28 (3H, d, *J* = 0.8 Hz), 3.20–3.30 (1H, m), 3.37–3.48 (1H, m), 3.59 (1H, dd, *J* = 12.9, 3.8 Hz), 3.70–3.80 (1H, m), 3.95 (1H, d, *J* = 12.7 Hz), 4.27 (1H, d, *J* = 12.4 Hz), 5.35 (1H, br s), 6.11 (1H, d, *J* = 0.9 Hz), 6.46 (2H, s), 6.54 (1H, td, *J* = 7.9, 1.7 Hz), 6.78 (1H, d, *J* = 1.3 Hz), 6.80–6.86 (1H, m), 7.01 (1H, d, *J* = 2.3 Hz), 7.16–7.30 (1H, m), 7.36 (1H, dd, *J* = 15.8, 2.3 Hz), 8.69 (1H, d, *J* = 3.5 Hz). ^13^C NMR (101 MHz, DMSO-*d*_6_): δ 11.7, 42.6, 46.1, 49.5, 51.6, 99.2 (d, *J* = 13.2 Hz), 101.2, 101.2 (d, *J* = 26.4 Hz), 102.2 (d, *J* = 2.2 Hz), 104.5 (d, *J* = 21.3 Hz), 105.0 (d, *J* = 26.4 Hz), 110.1 (d, *J* = 2.2 Hz), 130.4 (d, *J* = 10.3 Hz), 130.6 (d, *J* = 3.7 Hz), 142.3 (d, *J* = 2.2 Hz), 150.9 (d, *J* = 10.3 Hz), 152.7 (d, *J* = 10.3 Hz), 152.8 (d, *J* = 2.2 Hz), 158.8 (d, *J* = 250.2 Hz), 162.7, 163.3 (d, *J* = 239.9 Hz), 169.4. MS (ESI/APCI) *m*/*z*: 423.2 [M + H]^+^. Anal. calcd for C_22_H_20_F_2_N_6_O·0.3H_2_O: C, 61.76; H, 4.85; N, 19.64. Found: C, 61.94; H, 5.01; N, 19.42. Chiral HPLC analysis (CHIRALCEL OD-H 4.6 mm I.D. × 250 mm *L*, 5 μm with MeOH/diethylamine = 1000/1(v/v)): *t*_R_ = 10.0 min and 99% ee.

#### 6-Bromo-*N*-[(2,4-dimethoxyphenyl)methyl]isoquinolin-1-amine (**11b**)

A mixture of **9b** (827 mg, 3.41 mmol) and 2,4-dimethoxybenzylamine (2.56 mL, 17.1 mmol) was stirred at 120 °C for 3 h. The reaction mixture was diluted with EtOAc and washed with 10% KHSO_4_ aq. The organic layer was dried over MgSO_4_ and concentrated in vacuo. The residue was purified by column chromatography (NH silica gel, hexane/EtOAc = 95/5 to 50/50) to give the title compound (1.05 g, 83%) as pale yellow gum. ^1^H NMR (300 MHz, DMSO-*d*_6_): δ 3.72 (3H, s), 3.82 (3H, s), 4.61 (2H, d, *J* = 5.6 Hz), 6.41 (1H, dd, *J* = 8.4, 2.4 Hz), 6.56 (1H, d, *J* = 2.4 Hz), 6.85 (1H, d, *J* = 5.8 Hz), 7.07 (1H, d, *J* = 8.3 Hz), 7.62 (1H, dd, *J* = 8.9, 2.1 Hz), 7.82 (1H, br s), 7.85 (1H, d, *J* = 5.9 Hz), 7.98 (1H, d, *J* = 2.1 Hz), 8.29 (1H, d, *J* = 9.0 Hz). MS (ESI/APCI) *m*/*z*: 373.0, 375.0 (found).

#### 6-Bromo-*N*-[(2,4-dimethoxyphenyl)methyl]phthalazin-1-amine (**11c**)

A mixture of **9c** (1.50 g, 6.16 mmol) and 2,4-dimethoxybenzylamine (2.76 mL, 18.5 mmol) was stirred at 130 °C overnight. The mixture was purified by column chromatography (NH silica gel, hexane/EtOAc = 95/5 to 0/100) to give the title compound (1.20 g, 52%) as a brown solid. ^1^H NMR (300 MHz, DMSO-*d*_6_): δ 3.71 (3H, s), 3.72 (3H, s), 4.66–4.76 (2H, m), 6.85–6.95 (2H, m), 7.02–7.07 (1H, m), 8.00–8.08 (2H, m), 8.18–8.24 (1H, m), 8.27–8.35 (1H, m), 8.85–8.92 (1H, m). MS (ESI/APCI) *m*/*z*: 374.0, 376.0 (found).

#### *tert*-Butyl 4-(1-{[(2,4-Dimethoxyphenyl)methyl]amino}phthalazin-6-yl)piperazine-1-carboxylate (**12**)

A mixture of **11c** (1.00 g, 2.67 mmol), *tert*-butyl piperazine-1-carboxylate (0.995 g, 5.34 mmol), Pd(dba)_2_ (0.307 g, 0.534 mmol), RuPhos (0.100 g, 0.214 mmol), NaO^*t*^Bu (0.514 g, 5.34 mmol), and DME (8 mL) was stirred at 110 °C for 1 h under microwave irradiation. Water and EtOAc were added, and the solid was collected and washed with EtOAc to give the title compound (0.532 g, 42%) as a pale yellow solid. ^1^H NMR (300 MHz, DMSO-*d*_6_): δ 1.43 (9H, s), 3.33–3.40 (4H, m), 3.46–3.55 (4H, m), 3.72 (3H, s), 3.83 (3H, s), 4.64 (2H, d, *J* = 5.5 Hz), 6.42 (1H, dd, *J* = 8.4, 2.4 Hz), 6.57 (1H, d, *J* = 2.4 Hz), 7.09 (1H, d, *J* = 8.4 Hz), 7.16 (1H, d, *J* = 2.4 Hz), 7.47 (1H, t, *J* = 5.6 Hz), 7.59 (1H, dd, *J* = 9.2, 2.5 Hz), 8.20 (1H, d, *J* = 9.0 Hz), 8.69 (1H, s). MS (ESI/APCI) *m*/*z*: 480.3 [M + H]^+^.

#### *N*-[(2,4-Dimethoxyphenyl)methyl]-6-(piperazin-1-yl)phthalazin-1-amine Dihydrochloride (**13**)

A mixture of **12** (532 mg, 1.11 mmol), MeOH (10 mL), and 4 M HCl/EtOAc (10 mL) was stirred at room temperature for 2 h. The mixture was concentrated in vacuo. The residue was washed with EtOAc to give the title compound (450 mg, 90%) as a light brown solid. ^1^H NMR (300 MHz, DMSO-*d*_6_): δ 3.20–3.30 (4H, m), 3.77 (3H, s), 3.78–3.84 (7H, m), 4.67 (2H, d, *J* = 5.6 Hz), 6.52 (1H, dd, *J* = 8.3, 2.3 Hz), 6.63 (1H, d, *J* = 2.3 Hz), 7.18 (1H, d, *J* = 8.3 Hz), 7.55 (1H, d, *J* = 2.3 Hz), 7.78 (1H, d, *J* = 8.3 Hz), 8.60–8.77 (2H, m), 9.58 (2H, br s), 9.85 (1H, br s), 13.77 (1H, br s). MS (ESI/APCI) *m*/*z*: 380.3 [M + H-2HCl]^+^.

#### *tert*-Butyl 4-{1-[(2,4-Dimethoxybenzyl)amino]phthalazin-6-yl}-3,6-dihydropyridine-1(2*H*)-carboxylate (**14**)

A mixture of **11c** (600 mg, 1.60 mmol), *tert*-butyl 4-(4,4,5,5-tetramethyl-1,3,2-dioxaborolan-2-yl)-3,6-dihydropyridine-1(2*H*)-carboxylate (595 mg, 1.92 mmol), PdCl_2_(dppf) (117 mg, 0.160 mmol), Cs_2_CO_3_ (1.05 g, 3.21 mmol), DME (8 mL), and water (1 mL) was stirred at 120 °C under microwave irradiation for 1 h. Water and EtOAc were added, and the precipitate was collected and washed with MeOH to give the title compound (575 mg, 75%) as a light gray solid. ^1^H NMR (300 MHz, DMSO-*d*_6_): δ 1.44 (9H, s), 2.53–2.67 (2H, m), 3.59 (2H, t, *J* = 5.5 Hz), 3.72 (3H, s), 3.83 (3H, s), 4.07 (2H, br s), 4.67 (2H, d, *J* = 4.9 Hz), 6.36–6.53 (2H, m), 6.58 (1H, d, *J* = 2.3 Hz), 7.11 (1H, d, *J* = 8.3 Hz), 7.62–7.83 (1H, m), 7.91 (1H, s), 8.02 (1H, d, *J* = 8.3 Hz), 8.34 (1H, d, *J* = 8.7 Hz), 8.86 (1H, s). MS (ESI/APCI) *m*/*z*: 477.3 [M + H]^+^.

#### *N*-[(2,4-Dimethoxyphenyl)methyl]-6-(1,2,3,6-tetrahydropyridin-4-yl)phthalazin-1-amine Dihydrochloride (**15**)

A mixture of **14** (575 mg, 1.21 mmol), MeOH (5 mL), and 4 M HCl/EtOAc (5 mL) was stirred at room temperature for 2 h. The solid was collected and washed with EtOAc to give the title compound (478 mg, 88%) as a light gray solid. ^1^H NMR (300 MHz, DMSO-*d*_6_): δ 2.76–2.91 (2H, m), 3.44–3.73 (2H, m), 3.77 (3H, s), 3.79 (3H, s), 3.82–3.90 (2H, m), 4.72 (2H, d, *J* = 5.3 Hz), 6.51 (1H, dd, *J* = 8.3, 2.3 Hz), 6.61–6.74 (2H, m), 7.23 (1H, d, *J* = 8.3 Hz), 8.21–8.37 (2H, m), 8.88 (1H, d, *J* = 9.0 Hz), 9.00 (1H, br s), 9.59 (2H, br s), 10.26 (1H, br s), 14.20 (1H, br s). MS (ESI/APCI) *m*/*z*: 377.2 [M + H-2HCl]^+^.

#### *tert*-Butyl (3*R*)-4-(3-Fluorophenyl)-3-methylpiperazine-1-carboxylate (**(*R*)-17**)

A mixture of 1-bromo-3-fluorobenzene (0.255 mL, 2.29 mmol), **(*R*)-16** (458 mg, 2.29 mmol), Pd_2_(dba)_3_ (209 mg, 0.229 mmol), *rac*-BINAP (142 mg, 0.229 mmol), NaO^*t*^Bu (659 mg, 6.86 mmol), and toluene (5 mL) was stirred at 110 °C for 1.5 h under microwave irradiation. The reaction mixture was filtered through a pad of Celite, and the filtrate was concentrated in vacuo. The residue was purified by column chromatography (silica gel, hexane/EtOAc = 100/0 to 50/50) to give the title compound (224 mg, 36%) as an orange oil. ^1^H NMR (300 MHz, DMSO-*d*_6_): δ 0.91 (3H, d, *J* = 6.4 Hz), 1.42 (9H, s), 2.80–3.39 (4H, m), 3.70–4.13 (3H, m), 6.45–6.58 (1H, m), 6.63–6.77 (2H, m), 7.14–7.32 (1H, m). MS (ESI/APCI) *m*/*z*: 295.0 [M + H]^+^.

#### *tert*-Butyl (3*S*)-4-(3-Fluorophenyl)-3-methylpiperazine-1-carboxylate (**(*S*)-17**)

The title compound was prepared in 42% yield using **(*S*)-16** in an analogous manner to **(*R*)-17** as an orange oil. ^1^H NMR (300 MHz, DMSO-*d*_6_): δ 0.92 (3H, d, *J* = 6.4 Hz), 1.42 (9H, s), 2.80–3.23 (3H, m), 3.32–3.37 (1H, m), 3.64–4.10 (3H, m), 6.44–6.57 (1H, m), 6.61–6.78 (2H, m), 7.15–7.25 (1H, m). MS (ESI/APCI) *m*/*z*: 295.2 [M + H]^+^.

#### (2*R*)-1-(3-Fluorophenyl)-2-methylpiperazine Hydrochloride (**(*R*)-18**)

**(*R*)-17** (244 mg, 0.829 mmol) was dissolved in 4 M HCl/EtOAc (3 mL), after which the reaction was left to stir at room temperature for 3 h. The mixture was concentrated in vacuo. The residue was suspended in EtOAc/2-propanol/^*i*^Pr_2_O, and the mixture was concentrated in vacuo. The residue was washed with ^*i*^Pr_2_O to give the title compound (172 mg, 90%) as a light brown solid. ^1^H NMR (300 MHz, DMSO-*d*_6_): δ 1.11 (3H, d, *J* = 7.2 Hz), 2.95–3.34 (5H, m), 3.46–3.58 (1H, m), 4.15–4.29 (1H, m), 6.59–6.66 (1H, m), 6.75–6.83 (2H, m), 7.20–7.35 (1H, m), 9.12 (1H, br s), 9.61 (1H, br s). MS (ESI/APCI) *m*/*z*: 195.1 [M + H-HCl]^+^.

#### (2*S*)-1-(3-Fluorophenyl)-2-methylpiperazine Hydrochloride (**(*S*)-18**)

The title compound was prepared in 96% yield using **(*S*)-17** in an analogous manner to **(*R*)-18** as a light brown solid. 1H NMR (300 MHz, DMSO-*d*_6_): δ 1.10 (3H, d, *J* = 6.8 Hz), 2.94–3.32 (5H, m), 3.45–3.59 (1H, m), 4.14–4.27 (1H, m), 6.57–6.67 (1H, m), 6.74–6.81 (2H, m), 7.20–7.32 (1H, m), 9.01 (1H, br s), 9.49 (1H, br s). MS (ESI/APCI) *m*/*z*: 195.2 [M + H-HCl]^+^.

#### 1-Benzyl-3-(5-methyl-1,2-oxazol-3-yl)piperazine (**20**)

A mixture of **19** (1.17 mL, 4.65 mmol), 5-methylisoxazole-3-carbaldehyde (0.517 g, 4.65 mmol), and acetonitrile (18 mL) was stirred under nitrogen in the presence of 4 Å molecular sieves at room temperature overnight. After confirming the formation of the imine by TLC, the reaction mixture was filtered through Celite with acetonitrile. The solvent was removed in vacuo, after which the residue and [Ir(ppy)_2_(dtbpy)]PF_6_ (43.0 mg, 0.0465 mmol) were dissolved in a 25 mL solution of acetonitrile/2,2,2-trifluoroethanol (9/1(v/v)). The mixture was stirred for 3 h at room temperature under exposure to blue LED light (30 W, Penn photoreactor from Merck, 450 nm (fan on, light intensity of 100%)). Then, water (0.5 mL) was added to the reaction, which was stirred for 5 min. The reaction mixture was concentrated in vacuo, and the residue was purified by column chromatography (NH silica gel, hexane/EtOAc = 75/25 to 10/90) to give the title compound (0.750 g, 63%) as a yellow-orange solid. ^1^H NMR (300 MHz, DMSO-*d*_6_): δ 1.93–2.14 (2H, m), 2.31–2.39 (3H, m), 2.60–2.81 (3H, m), 2.82–2.92 (1H, m), 3.40–3.57 (2H, m), 3.73–3.87 (1H, m), 6.12–6.25 (1H, m), 7.07–7.42 (6H, m). MS (ESI/APCI) *m*/*z*: 258.2 [M + H]^+^.

#### 4-Benzyl-1-(3-fluorophenyl)-2-(5-methyl-1,2-oxazol-3-yl)piperazine (**21**)

A mixture of **20** (720 mg, 2.80 mmol), 1-bromo-3-fluorobenzene (0.338 mL, 3.08 mmol), Pd(dba)_2_ (322 mg, 0.560 mmol), DavePhos (88.0 mg, 0.224 mmol), NaO^*t*^Bu (538 mg, 5.60 mmol), and DME (12 mL) was stirred at 110 °C under microwave irradiation for 2 h. The reaction mixture was filtered and washed with MeOH. The mixture was concentrated in vacuo. To the residue were added water and EtOAc, and the mixture was extracted with EtOAc. The combined organic layer was washed with brine, dried over Na_2_SO_4_, and concentrated in vacuo. The residue was purified by column chromatography (NH silica gel, hexane/EtOAc = 75/25 to 0/100) to give the title compound (365 mg, 37%) as a yellow oil. ^1^H NMR (300 MHz, DMSO-*d*_6_): δ 2.17–2.30 (1H, m), 2.33 (3H, d, *J* = 0.8 Hz), 2.52–2.55 (1H, m), 2.85–3.07 (2H, m), 3.12–3.29 (1H, m), 3.46–3.60 (3H, m), 5.06–5.21 (1H, m), 6.12 (1H, d, *J* = 0.8 Hz), 6.42–6.58 (1H, m), 6.62–6.77 (2H, m), 7.08–7.20 (1H, m), 7.21–7.39 (5H, m). MS (ESI/APCI) *m*/*z*: 352.1 [M + H]^+^.

#### *tert*-Butyl 4-(3-Fluorophenyl)-3-(5-methyl-1,2-oxazol-3-yl)piperazine-1-carboxylate (**22**)

To a solution of **21** (360 mg, 1.02 mmol) in acetonitrile (10 mL) was added 1-chloroethyl chloroformate (0.133 mL, 1.23 mmol). The reaction was stirred at room temperature for 3 h, after which the reaction mixture was evaporated to dryness. MeOH (10 mL) was added to the reaction vessel, after which the reaction mixture was stirred at room temperature overnight and was subsequently heated under reflux for 3 h. The solvent was again removed in vacuo, and the crude mixture was dissolved in THF (20 mL). Et_3_N (0.171 mL, 1.23 mmol) was added followed by Boc_2_O (0.285 mL, 1.23 mmol). The reaction mixture was stirred at room temperature overnight. The reaction mixture was evaporated to dryness, and the residue was purified by column chromatography (silica gel, hexane/EtOAc = 90/10 to 50/50) to give the title compound (342 mg, 92%) as a yellow oil. ^1^H NMR (300 MHz, DMSO-*d*_6_): δ 1.30 (9H, br s), 2.32 (3H, s), 3.15–3.30 (2H, m), 3.37–4.21 (4H, m), 5.11–5.23 (1H, m), 6.02 (1H, d, *J* = 0.8 Hz), 6.39–6.60 (1H, m), 6.64–6.79 (2H, m), 7.07–7.28 (1H, m). MS (ESI/APCI) *m*/*z*: 306.2 [M + H-^*t*^Bu]^+^.

#### 1-(3-Fluorophenyl)-2-(5-methyl-1,2-oxazol-3-yl)piperazine (**23**)

A mixture of **22** (342 mg, 0.946 mmol) and 4 M HCl/EtOAc (10 mL) was stirred at room temperature overnight. The solvent was removed in vacuo, after which the crude material was dissolved in acetonitrile (12 mL) and eluted through carbonate ion exchange resin. The filtrate was concentrated in vacuo to give the title compound (218 mg, 88%) as a pale yellow oil. ^1^H NMR (300 MHz, DMSO-*d*_6_): δ 2.31 (3H, d, *J* = 0.8 Hz), 2.67–2.84 (1H, m), 2.93–3.23 (4H, m), 3.32–3.42 (2H, m), 5.01 (1H, t, *J* = 3.1 Hz), 6.06 (1H, d, *J* = 0.8 Hz), 6.37–6.57 (1H, m), 6.59–6.78 (2H, m), 7.06–7.25 (1H, m). MS (ESI/APCI) *m*/*z*: 262.1 [M + H]^+^.

#### (2*R*)-1-(3-Fluorophenyl)-2-(5-methyl-1,2-oxazol-3-yl)piperazine (**(*R*)-23**)

**23** (218 mg, 0.83 mmol) was optically resolved by chiral preparative HPLC (CHIRALPAK IC, 50 mm I.D. × 500 mm *L*, 20 μm with hexane/EtOH/diethylamine = 600/400/1(v/v/v)). The second eluting fraction was collected and concentrated in vacuo to give **(*R*)-23** (97.8 mg, 45%) as a pale yellow oil. MS (ESI/APCI) *m*/*z*: 262.1 [M + H]^+^. Chiral HPLC analysis (CHIRALPAK IC 4.6 mm I.D. × 250 mm *L*, 5 μm with hexane/EtOH/diethylamine = 600/400/1(v/v/v)): *t*_R_ = 7.73 min and 99% ee.

#### 4-Bromo-2-(dimethoxymethyl)-6-fluorobenzonitrile (**25**)

To a solution of **24** (4.00 g, 17.5 mmol) in MeOH (80 mL) were added trimethoxymethane (9.60 mL, 87.7 mmol) and *p*-toluenesulfonic acid monohydrate (0.667 g, 3.51 mmol) at room temperature. The mixture was stirred at 60 °C for 1 h. The mixture was concentrated in vacuo. The residue was purified by column chromatography (silica gel, hexane/EtOAc = 100/0 to 50/50) to give the title compound (4.43 g, 92%) as a light brown solid. ^1^H NMR (300 MHz, DMSO-*d*_6_): δ 3.35 (6H, s), 5.57 (1H, s), 7.66 (1H, dd, *J* = 1.7, 0.8 Hz), 8.00 (1H, dd, *J* = 8.9, 1.8 Hz). MS (ESI/APCI) *m*/*z*: 276.0 (found).

#### 2-(Dimethoxymethyl)-6-fluoro-4-[4-(3-fluorophenyl)-3-(5-methyl-1,2-oxazol-3-yl)piperazin-1-yl]benzonitrile (**26**)

A mixture of **25** (273 mg, 1.00 mmol), **23** (200 mg, 0.765 mmol), Pd(dba)_2_ (88.0 mg, 0.153 mmol), RuPhos (28.6 mg, 0.0612 mmol), 8 M NaOH aq. (0.0956 mL, 0.765 mmol), and DME (4 mL) was stirred at 120 °C under microwave irradiation for 2 h. The mixture was concentrated in vacuo. The residue was purified by column chromatography (NH silica gel, hexane/EtOAc = 100/0 to 60/40) to give the title compound (250 mg, 72%) as a pale yellow amorphous powder. ^1^H NMR (300 MHz, DMSO-*d*_6_): δ 2.28 (3H, d, *J* = 0.8 Hz), 3.33 (6H, s), 3.34–3.55 (2H, m), 3.59–3.78 (2H, m), 3.82–3.95 (1H, m), 4.17–4.30 (1H, m), 5.20–5.34 (1H, m), 5.40 (1H, s), 6.06 (1H, d, *J* = 0.9 Hz), 6.48–6.58 (1H, m), 6.72 (1H, s), 6.73–6.78 (1H, m), 6.83 (1H, d, *J* = 2.3 Hz), 6.92 (1H, dd, *J* = 13.6, 2.3 Hz), 7.14–7.27 (1H, m). MS (ESI/APCI) *m*/*z*: 477.2 [M + Na]^+^.

#### 2-(Dimethoxymethyl)-6-fluoro-4-[(3*R*)-4-(3-fluorophenyl)-3-(5-methyl-1,2-oxazol-3-yl)piperazin-1-yl]benzonitrile (**(*R*)-26**)

A mixture of **25** (107 mg, 0.390 mmol), **(*R*)-23** (66.6 mg, 0.255 mmol), Pd(dba)_2_ (29.3 mg, 0.0510 mmol), RuPhos (9.52 mg, 0.0204 mmol), NaO^*t*^Bu (49.0 mg, 0.510 mmol), and DME (2 mL) was stirred at 120 °C under microwave irradiation for 1.5 h. The mixture was purified by column chromatography (silica gel, hexane/EtOAc = 100/0 to 60/40) to give the title compound (82.3 mg, 71%) as a pale yellow oil. ^1^H NMR (300 MHz, DMSO-*d*_6_): δ 2.28 (3H, d, *J* = 0.8 Hz), 3.24–3.51 (8H, m), 3.61–3.79 (2H, m), 3.83–3.94 (1H, m), 4.19–4.29 (1H, m), 5.26–5.33 (1H, m), 5.40 (1H, s), 6.06 (1H, d, *J* = 0.8 Hz), 6.44–6.63 (1H, m), 6.69–6.79 (2H, m), 6.83 (1H, d, *J* = 2.3 Hz), 6.92 (1H, dd, *J* = 13.5, 2.3 Hz), 7.13–7.30 (1H, m). MS (ESI/APCI) *m*/*z*: 441.2 (found).

### Single-Crystal X-ray Structural Analysis

Crystal data for **(*S*)-8**: C_22_H_21_F_2_N_6_O^+^·C_20_H_17_O_10_·H_2_O, MW = 858.81; crystal size, 0.11 × 0.06 × 0.05 mm; colorless, block; monoclinic, space group *P*2_1_, *a* = 7.63959(5) Å, *b* = 12.43180(8) Å, *c* = 20.92670(13) Å, α = γ = 90°, β = 96.5053(6)°, *V* = 1974.69(2) Å^3^, *Z* = 2, *Dx* = 1.444 g/cm^3^, *T* = 100 K, μ = 0.963 mm^–1^, λ = 1.54184 Å, *R*_1_ = 0.0279, *wR*_2_ = 0.0737, *S* = 1.022, Flack parameter^23^ = −0.02(3).

All measurements were made on a Rigaku XtaLAB P200 diffractometer using multilayer mirror monochromated Cu Kα radiation. The structure was solved by direct methods with SHELXT-2018/2^24^ and was refined using full-matrix least-squares on *F*^2^ with SHELXL-2018/3.^25^ All non-H atoms were refined with anisotropic displacement parameters. CCDC 2251422 for compound **(*S*)-8** contains the supplementary crystallographic data for this paper. These data can be obtained free of charge from The Cambridge Crystallographic Data Centre via http://www.ccdc.cam.ac.uk/structures.

### Vector Construction, Expression, and Purification of Recombinant Human C1s Protein

The sequence of human C1s comprising amino acids 358–688 with mutation of Glu518 to Ala518 was gene synthesized and cloned into the pFastBac1 vector from Invitrogen. Recombinant baculovirus for human C1s (358–688, E518A) was generated using a Bac to Bac baculovirus expression system (Invitrogen) and amplified in High Five insect cells (Invitrogen). The protein was expressed as a gp67 signal peptide fusion protein in ESF 921 medium (Expression Systems). Growth medium was collected, and protein concentration and diafiltration with 50 mM Tris, pH 7.9, 50 mM NaCl, and 0.25 mM TCEP exchange buffer were performed using a 10 kDa regenerated cellulose membrane.

The C1s protein was purified using a HiTrap Q column (Qiagen) with a salt gradient from 0 to 500 mM NaCl followed by Superdex-200 chromatography (GE Healthcare). The fractions that contained C1s protein were further purified with a heparin affinity column (Qiagen) with a salt gradient from 0 to 500 mM NaCl, as well as a PD-10 desalting column (GE Healthcare) with buffer 50 mM triethanolamine HCl pH 7.4 and 145 mM NaCl. The resulting C1s protein was activated by C1r cleavage and then concentrated to ∼10 mg/mL with centrifugal filters (Amicon Ultra-15, 10 kDa cutoff). The protein concentration was estimated using a NanoDrop2000 instrument (Thermo Fisher Scientific).

Fractions containing the protein of interest were pooled, concentrated to ∼2 mg/mL, and flash-frozen in liquid nitrogen for storage at −80 °C. For compound cocrystallization experiments, an aliquot of the frozen protein was incubated with the compound (final concentration of 5 mM) at 4 °C overnight and then further concentrated to 10 mg/mL.

### X-ray Structure Analysis

Crystals suitable for data collection were obtained by vapor diffusion in sitting drops at 4 °C. Reservoirs contained 18–24% PEG 3350, 50–100 mM ammonium sulfate, and 100 mM Tris pH 7.5–8.0. Crystals that took 40–50 days to grow were immersed in a mother liquor solution containing 22% ethylene glycol for cryoprotection and flash frozen in liquid nitrogen. Crystals of the C1s complex grew in the triclinic space group *P*1 and contained four molecules in the asymmetric unit.

Diffraction data to 2.60 Å were collected from single cryogenically protected crystals at the beam line 5.0.3 of the Advanced Light Source at Lawrence Berkeley National Laboratory. Data were reduced using the XDS software package.^26^ The structure was determined by molecular replacement with either MOLREP^27^ or PHASER^28^ of the CCP4 program suite and refined with the program REFMAC.^29^ Several cycles of model building with COOT^30^ and refinement were performed for improving the quality of the model. The coordinates and structure factors were deposited in the Protein Data Bank with the accession code 8GMN.

### Protein Preparation for In Silico Calculation

The protein structures of PDB entries 1ELV and 8GMN were used for docking calculation. Preparatory calculation of the protein structure was performed with Protein Preparation wizard in Maestro12.7 (Schrödinger 2021-1) (Schrödinger, LLC; New York, USA). Regarding the structure of the PDB entry 8GMN, the peptidic residues Ser364-Asn367, Thr385-Tyr392, and Cys421-Glu434 were removed, after which the N-terminus and C-terminus except for Ile438 were capped with acetyl or *N*-methyl groups for hot spot prediction by mixed-solvent molecular dynamics (MxMD). Hydrogen atoms were added, and optimal ionization states were assigned under pH = 7.0 ± 2.0. All water molecules observed in the crystal structure were removed. Restrained minimization that allows atoms to move within 0.3 Å of RMSD against the positions in the original crystal structure was performed with the OPLS3 force field.

### Docking Study

The compounds were prepared to add hydrogens and assign optimal ionization states using LigPrep in Maestro12.7. The prepared compounds were docked into the protein structure using Glide9.0 in the standard precision (SP) mode to obtain up to 5 docking poses, which were refined by energy minimization with the OPLS force field including the flexible amino acid residues within 5.5 Å of the compound. The pose with the lowest prime energy was selected as the presumed binding pose.

### Hot Spot Prediction

MxMD calculation was performed using the mxmd program of Schrödinger2021-1 in default setting except for the kinds of probes. Dimethylsulfoxide, formamide, oxiranemethanol, thiazole, and pyridine were used as probes.

### p*K*_a_ Prediction

p*K*_a_ prediction was performed using Jaguar (Schrödinger 2021-1) in default setting.

### Human C1s Enzyme Assay

Recombinant human C1s (358–688 aa) fused with both the N-terminal IgG signal peptide and the C-terminal His tag was expressed in HEK293 cells and purified. The assay was performed in a 384-well clear plate in a total volume of 20 μL of an assay buffer 1 [50 mmol/L Tris–HCl (pH 7.5), 150 mmol/L NaCl, 0.01% (w/v) Tween 20, and 0.01% (w/v) BSA]. Compounds were dissolved in DMSO and diluted to various concentrations. After 20 nL of compound solution was added to the plate, 10 μL of substrate solution containing a 250 μmol/L Z-Lys-SBzl peptide (Watanabe Chemical, Hiroshima, Japan) and 250 μmol/L DTNB (5,5′-dithiobis(2-nitrobenzoic acid)) (Sigma, MO, USA) was added to the plate. Then, 8 nmol/L human C1s was added to the plate and incubated at room temperature for 60 min. The absorbance at 405 nm was detected using an EnVision multilabel plate reader. The percentage of inhibition was calculated relative to control wells containing no enzyme (100% inhibition) and control wells containing no inhibitor (0% inhibition). The IC_50_ values were calculated using IDBS XLfit software.

### Mouse C1s Enzyme Assay

Recombinant mouse C1s (22–694 aa) fused with both the N-terminal IgG signal peptide and the C-terminal His tag was expressed in Expi293 cells and purified. Mouse C1s was activated by recombinant human C1r prior to use. Mouse C1s assay conditions were the same as for human C1s, except that the enzyme solution concentration was 30 nmol/L.

### Human C1r Enzyme Assay

Recombinant human C1r (375–705 aa) fused with both the N-terminal IgG signal peptide and the C-terminal His tag was expressed in HEK293 cells and purified. Human C1r assay conditions were the same as for human C1s, except that the enzyme solution concentration was 3 nmol/L and the substrate peptide was 250 μmol/L Z-Gly-Arg-SBzl (custom synthesis from Peptide Institute, Osaka, Japan) instead of Z-Lys-SBzl.

### Human MASP2 Enzyme Assay

Recombinant human MASP2 (298–686 aa) fused with both the N-terminal IgG signal peptide and the C-terminal His tag was expressed in Expi293 cells and purified. The assay was performed in a 384-well black plate in a total volume of 6 μL of the assay buffer 1. After 20 nL of compound solution was added to the plate, 3 μL of substrate solution containing a 20 μmol/L Z-Gly-Arg-SBzl peptide and 20 μmol/L BES-Thio (FUJIFILM Wako, Osaka, Japan) was added to the plate. Then, 0.6 nmol/L human MASP2 was added to the plate and incubated at room temperature for 60 min. The fluorescence at an excitation wavelength of 485 nm and an emission wavelength of 535 nm was detected using an EnVision multilabel plate reader.

### Human Factor D Enzyme Assay

Recombinant human factor D (26–253 aa) fused with both the N-terminal IgG signal peptide and the C-terminal His tag was expressed in Expi293 cells and purified. The human factor D assay condition was the same as for human MASP2, except that the enzyme solution concentration was 20 nmol/L and the substrate solution contained a 400 μmol/L Z-Lys-SBzl peptide and 50 μmol/L BES-Thio.

### Human Thrombin Enzyme Assay

The human thrombin enzyme activity was determined by a SensoLyte 520 thrombin activity assay kit (AnaSpec, CA, USA). Purified human thrombin and the substrate were diluted in the assay buffer 1. After 20 nL of compound solution was added to a 384-well black plate, 3 μL of the 2 μmol/L 5-FAM/QXLTM 520 thrombin substrate was added to the plate. Then, 8 nmol/L human thrombin was added to the plate and incubated at room temperature for 60 min. The fluorescence at an excitation wavelength of 485 nm and an emission wavelength of 535 nm was detected using an EnVision multilabel plate reader.

### Human Trypsin Enzyme Assay

Recombinant human trypsin (FUJIFILM Wako, Osaka, Japan) and the substrate were diluted in an assay buffer [50 mmol/L Tris–HCl (pH 7.5), 145 mmol/L NaCl, 2 mmol/L CaCl_2_, and 0.01% (w/v) Tween 20]. After 20 nL of compound solution was added to a 384-well black plate, 3 μL of substrate solution containing 25 μmol/L Boc-Phe-Ser-Arg-MCA (Peptide Institute, Osaka, Japan) was added to the plate. Then, 6 mU/mL human trypsin was added to the plate and incubated at room temperature for 60 min. The fluorescence at an excitation wavelength of 360 nm and an emission wavelength of 460 nm was detected using an EnVision multilabel plate reader.

### Human Kallikrein Enzyme Assay

Recombinant human kallikrein (R&D Systems, MN, USA) was activated by thermolysin (R&D Systems, MN, USA) prior to use. Compounds were dissolved in DMSO and then diluted in an assay buffer [50 mmol/L Tris–HCl (pH 7.5), 150 mmol/L NaCl, 10 mmol/L CaCl_2_, and 0.01% (w/v) Tween 20]. After 5 μL of compound solution was added to a 384-well black plate, 5 μL of substrate solution containing 150 μmol/L Pro-Phe-Arg-AMC (Peptide Institute, Osaka, Japan) was added to the plate. Then, 0.42 nmol/L human kallikrein was added to the plate and incubated at room temperature for 60 min. The fluorescence at an excitation wavelength of 380 nm and an emission wavelength of 460 nm was detected using a SpectraMax Paradigm plate reader.

### Human Plasmin Enzyme Assay

Recombinant human plasmin (Zedira, Hesse, Germany) and the substrate were diluted in an assay buffer [50 mmol/L Tris–HCl (pH 7.5), 150 mmol/L NaCl, and 0.01% (w/v) Tween 20]. Compounds were dissolved in DMSO and then diluted in an assay buffer. After 5 μL of compound solution was added to a 384-well black plate, 5 μL of substrate solution containing 75 μmol/L Boc-Val-Leu-Lys-MCA (Peptide Institute, Osaka, Japan) was added to the plate. Then, 11.25 nmol/L human plasmin was added to the plate and incubated at room temperature for 60 min. The fluorescence at an excitation wavelength of 380 nm and an emission wavelength of 460 nm was detected using an EnVision multilabel plate reader.

### C5b-9 MAC Complex Formation Study

The levels of the complement activation product C5b-9 MAC were determined by a Wieslab complement system classical pathway kit (Svar Life Science, Malmo, Sweden). Briefly, diluted human serum was preincubated with the reagent diluent containing 0.3% DMSO or a range of concentrations of C1s inhibitors for 10 min at room temperature. Then, the preincubated serum was added to each well, and the plate was incubated for 30 min at 37 ° C with a lid. The plate was then washed three times, and subsequent processes were conducted by following the manufacturer’s instructions. Lastly, the absorbance was measured with a Multiskan microplate photometer (Thermo Fisher Scientific, MA, USA), and the levels of complement activation were calculated based on the standard curve generated with calibrators with serially diluted human serum. The samples with serum treatment were set as 100%, and the activities of the negative control (NC) without the addition of serum were set as 0%.

### PAMPA

A PAMPA Evolution system (pION) was applied for the assay. After incubating the test compound (10 μmol/L) for 3 h at 25 °C, the permeation coefficient value was calculated with determining the test compound by LC/MS/MS.

### Evaluation of Membrane Permeability with Human Multidrug Resistance 1 (MDR-1) Expressing Cells

The transcellular transport study was performed as reported previously.^31^ In brief, the cells were grown in an HTS Transwell 96-well permeable support (pore size of 0.4 μm, 0.143 cm^2^ surface area) with a polyethylene terephthalate membrane (Corning Life Sciences, Lowell, MA, USA) at a density of 1.125 × 10^5^ cells/well. The cells were preincubated with M199 at 37 °C for 30 min. Subsequently, transcellular transport was initiated by the addition of M199 to apical compartments containing 1 μmol/L test compounds and terminated by the removal of each assay plate after 2 h. The aliquots in the opposite compartments were subjected to measurement for compound concentration by LC–MS/MS. Permeability was calculated using the permeated compound concentration.

### Animal Experiments

All animal experiments were performed in compliance with the Guidelines for the Care and Use of Laboratory Animals of Takeda Pharmaceutical Company Ltd.

### Pharmacokinetics Analysis in Mice

Male C57BL/6J mice were purchased from The Jackson Laboratory Japan, Inc. (Kanagawa, Japan). All animals were with free access to food and water prior to the treatment and were healthy throughout the experimental period. The compound was dissolved in 10% DMSO/10% Cremophor EL/20% PEG400/60% 0.1 mol/L citric acid solution and administered orally at doses of 10, 30, or 100 mg/kg. Blood samples were collected into heparinized tubes at the designated time points (0.5, 1, 2, 4, 8, and 24 h postdosing), and brains were also collected at 2 and 24 h postdosing. Plasma was separated from the blood samples by centrifugation. The plasma and brain concentrations were quantified with high-performance liquid chromatography–tandem mass spectrometry. The lower limit of quantitation was 1 ng/mL for plasma and 5 ng/mL for brains.

## Supporting Information Available

Coordinate file for the docking model of compound **3** and C1s (PDB)
Coordinate file for the docking model of compound **5g** and C1s (PDB)
Coordinate file for the docking model of compound **(*R*)-7** and C1s (PDB)
HPLC traces of compounds **4e**, **5g**, **(*R*)-7**, and **(*R*)-8** (PDF)
Molecular formula strings (CSV)

## Supplementary Material

jm3c00348_si_001.pdb

jm3c00348_si_002.pdb

jm3c00348_si_003.pdb

## Abbreviations

AUC: area under the curve
*rac*-BINAP: racemic 2,2′-bis(diphenylphosphino)-1,1′-binaphthyl
Boc_2_O: di-*tert*-butyl dicarbonate
DavePhos: 2-dicyclohexylphosphino-2-(*N*,*N*-dimethylamino)biphenyl
DIPEA: *N*,*N*-diisopropylethylamine
MAC: membrane attack complex
MASP2: mannan-binding lectin serine protease 2
MDR-1: multidrug resistance 1
MRT: mean residence time
PAMPA: parallel artificial membrane permeability assay
RuPhos: 2-dicyclohexylphosphino-2′,6′-diisopropoxybiphenyl
