# Supplementary material for: Discovery of a Novel Series of Potent, Selective, Orally Available, and Brain-Penetrable C1s Inhibitors for Modulation of the Complement Pathway
Source: J Med Chem. 2023 Apr 25;66(9):6354–71. doi: 10.1021/acs.jmedchem.3c00348 (PMC10184130; doi:10.1021/acs.jmedchem.3c00348)
Supplement: Supplementary file 4 — jm3c00348_si_004.pdf [file jm3c00348_si_004.pdf]

Supporting Information

Discovery of a Novel Series of Potent, Selective, Orally Available, and Brain Penetrable C1s Inhibitors for Modulation of the Complement Pathway

Zenichi Ikeda,[*,†] *Taku Kamei,*[†] *Yusuke Sasaki,*[†] *Matthew Reynolds,*[†] *Nozomu Sakai,*[†] *Masato Yoshikawa,*[†] *Michiko Tawada,*[†] *Nao Morishita,*[†] *Douglas R. Dougan,*[‡] *Chien-Hung Chen,*[‡] *Irena Levin,*[‡] *Hua Zou,*[‡] *Masako Kuno,*[†] *Naoto Arimura,*[†] *Yusuke Kikukawa,*[†] *Mitsuyo Kondo,*[§] *Kimio Tohyama,*[†] *and Kenjiro Sato* [†]

[†] *Research, Takeda Pharmaceutical Company Ltd., 26-1, Muraokahigashi 2-chome, Fujisawa, Kanagawa 251-8555, Japan.*

[‡] *Structural Biology, Takeda Development Center Americas, Inc., San Diego, California, United States 92121.*

[§] *Discovery Biology, Discovery Science, Axcelead Drug Discovery Partners, Inc., 26-1, Muraoka-Higashi 2-chome Fujisawa, Kanagawa 251-0012, Japan.*

Corresponding Author

* Correspondence should be addressed to:

Zenichi Ikeda: E-mail: zenichi.ikeda@takeda.com

Table of contents

Analytical HPLC traces S2

Analytical HPLC traces

Compound **4e**

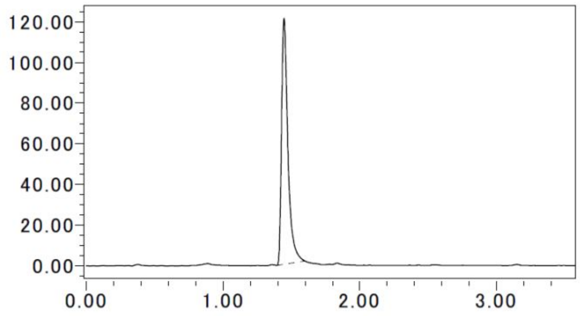

Result of Analysis

	Retention Time (min)	Peak area (μV×sec)	Divisional method	% of Area
1	1.446	390515	BB	100.00

Compound **5g**

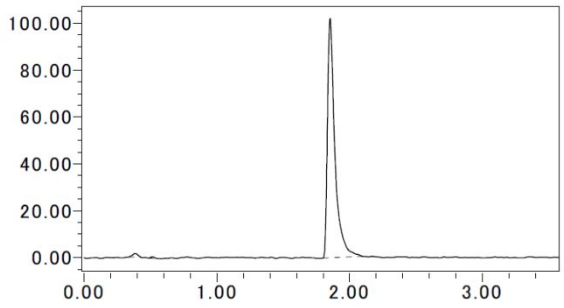

Result of Analysis

	Retention Time (min)	Peak area (μV×sec)	Divisional method	% of Area
1	0.383	4628	bb	1.14
2	0.512	1286	bb	0.32
3	1.853	397917	Bb	98.39
4	2.172	588	bb	0.15

Compound **(*R*)-7**

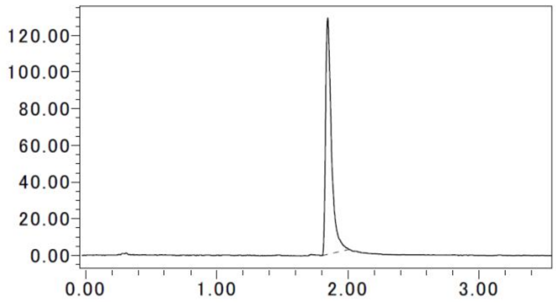

Result of Analysis

	Retention Time (min)	Peak area (μV×sec)	Divisional method	% of Area
1	1.846	403024	BB	100.00

Compound **(*R*)-8**

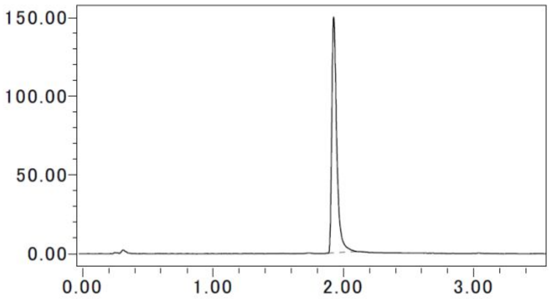

Result of Analysis

	Retention Time (min)	Peak area (μV×sec)	Divisional method	% of Area
1	1.926	398402	Bb	100.00
